# Effects of Structural and Electrical Defects on Vibration Suppression and Energy Harvesting Performance of Piezoelectric Metamaterial Beam

**DOI:** 10.3390/mi17101114

**Published:** 2026-09-24

**Authors:** Hongyan Wang, Xinru Jin, Guanglei Wang, Xiangwei Sun

**Affiliations:** College of Mechatronic Engineering, Qiqihar University, Qiqihar 161006, China

**Keywords:** metamaterial beam, piezoelectric local resonators, structural defects, electrical defects, vibration suppression, energy harvesting

## Abstract

This paper investigates the vibration suppression and energy harvesting performance of a locally resonant piezoelectric metamaterial beam (PMMB) with structural and electrical defects. The unit cell of a PMMB comprises a square aluminum substrate, two rubber plates sandwiching a piezoelectric plate, and a cylindrical stub on top. The structural and electrical defects are introduced by varying the material density of a stub, adjusting the spacing between the two adjacent piezoelectric local resonators (PLRs), and connecting inductors to selected PLRs, respectively. To investigate the theoretical band gap (BG), the effective BG (i.e., attenuation band), and the energy harvesting performance of defect-configured PMMB, supercell finite element were performed to obtain dispersion curves, while the six-PLR PMMB simulations were conducted to acquire transmission spectra and responding voltage outputs. Three distinct mechanisms are explored to enhance the performance of PMMB: defect states inside the effective BG for energy localization, defect–boundary interference for energy redistribution in the passband, and LC resonance in the shunted piezoelectric resonators for frequency-tunable energy harvesting. The results demonstrate that either stub-mass-reduced defect or substrate-length-increased spacing defect yield high voltages via energy localization in the effective BG, but they also narrow the effective BG. In contrast, tuning the center-to-center spacing of the two adjacent PLRs (near the excitation end) to an appropriate distance generates a dominant passband peak. The significant rises in both the far-end PLR voltage and the total voltage of the six PLRs clearly indicate energy redistribution within the passband. By selecting appropriate inductor parameters, LC-resonance-based electrical defects enable frequency-tunable voltage enhancement either at the effective BG edge or within the passband. Overall, this study provides comprehensive insight into how structural and electrical defects affect the response characteristics of the locally resonant PMMB.

## 1. Introduction

Acoustic-elastic metamaterials (AEMMs), which are also called locally resonant metamaterials or locally resonant phononic crystals (PCs) by some researchers, are artificially engineered periodic structures with unique properties that are not found in natural materials [1]. One of the most attractive features of AEMMs is the ability to restrict the acoustic/elastic wave transmission over a specified frequency range, which is termed a band gap (BG). Different from Bragg scattering BGs generated by conventional PCs, the operating principle of AEMMs is based on the local resonant effect induced by the resonators. Due to the existence of the local resonant BGs, AEMMs can break through the size limitation of PCs and then be used in microstructures. Hence, AEMMs have extensive potential applications in many fields such as vibration/noise control [2], cloaking [3], and superlens [4]. Piezoelectric materials, characterized by electromechanical coupling, enable energy conversion between mechanical and electrical domains, which underpins their widespread use in vibration control and energy harvesting [1,5]. The recent decade has witnessed a surge of interest in piezoelectric AEMMs, with research efforts covering three main categories: vibration suppression alone, energy harvesting alone, and the dual-functional integration of both in a single system [6,7,8].

Local resonant BGs, and their broadband counterparts, can be realized by tailored design of mechanical or electromechanical resonators attached to a host structure, with broadband BG strategies including multi-degree-of-freedom local resonators [9], acoustic black holes [10], negative-stiffness elements [11], and graded/disorder configurations [12,13]. In addition to mechanical tailoring of passive metamaterials, considerable attention has been devoted to the role of shunt circuits. While the conventional resistive-inductive shunt circuit is widely employed, it often necessitates a large inductance to realize low-frequency local resonance [14]. Alternative circuit topologies, such as negative capacitance shunts and synchronized charge extraction (SCE) circuits, have also been investigated. Negative capacitance circuits are particularly effective for extremely low-frequency vibration control [15,16]. For SCE circuits, studies on PMM plates with local resonators have demonstrated that inserting an inductor in parallel with the clamped capacitance of the piezoelectric patch significantly improves energy harvesting performance when the inductor-capacitor resonant frequency matches the excitation frequency [17].

Structural defects in phononic crystals and metamaterials have been extensively studied in various forms. A localized defect mode can be generated within the BG at a designated frequency by introducing a defect that breaks the periodicity of a conventional metamaterial or PC. From an energy harvesting perspective, the primary benefit of this defect mode lies in its ability to concentrate vibrational energy at the defect location, yielding a high-density input for the energy harvester [18]. The exploitation of defect modes for piezoelectric energy harvesting in metamaterials/PCs, whether with single or multiple defects, has attracted considerable research attention. Wang et al. [19] investigated acoustic energy harvesting using the piezoelectric curved beams in the cavity of the PC with a defect. The introduction of the defect gives rise to a defect band corresponding to the defect mode within the PC structure. The defect band acts as a passband in the BG, and it leads to the localization of acoustic waves at the defect site at the defect-mode frequency. Consequently, the maximum energy is achieved at that frequency via the piezoelectric beams. Deng et al. [20] presented a magneto-elastic planar PC with a defect, where an embedded piezoelectric transducer is used to convert the vibration energy into electrical energy. By varying the applied magnetic field and the pre-stress, the BGs and the defect bands of the in-plane modes are tuned, and thus a wide range of vibration frequencies and more broadly distributed vibration energy is obtained. Hyun et al. [21] designed an attachable locally resonant metamaterial with a defect-induced BG to localize and amplify low-frequency flexural vibrations, and integrate a piezoelectric harvester within the defect for simultaneous vibration isolation and energy harvesting. Experimental results demonstrate a 43-fold enhancement in output power over a broad frequency range (310–380 Hz) compared to a bare plate. Jo et al. [22] investigated the effects of the supercell size and the defect location on the energy localization and harvesting performance of a PC-based piezoelectric energy harvesting system. It was found that increasing supercell size enhances the energy harvesting performance. The optimal defect location is near the first layer of the supercell structure, in which the maximum piezoelectric energy is obtained. Hao et al. [23] presented a locally resonant metamaterial sandwich plate with cantilever spiral beam-mass resonators and various piezoelectric defects, and analytically and numerically investigated its BG characteristics and energy harvesting performance, revealing that two-point defects offer superior energy concentration and capture efficiency at high resistance, while increasing defect numbers disperses energy localization and reduces harvesting efficacy. Existing studies have largely focused on conventional mechanical defects, such as the removal, replacement, or alteration of resonators, as well as their selective substitution with PZT-layered counterparts. Despite this body of work, variations in PLR material density, positional spacing irregularities, and electrical defects have rarely been systematically evaluated on the same locally resonant PMMB structure, particularly in the context of the combined functionality of vibration suppression and energy harvesting.

In this paper, we systematically investigate three types of defects: mass, spacing, and electrical, within a unified PMMB framework. Dispersion curves are calculated using the finite element method with the supercell technique, while the transmission spectra and voltage outputs are obtained from finite element simulations of the six-PLR PMMB. The effects of these defects on the theoretical BGs, effective BGs (i.e., attenuation band), and voltage outputs are systematically studied, revealing distinct mechanisms including energy localization, global energy redistribution, and frequency-tunable electrical resonance. This study provides a comprehensive understanding of how structural and electrical defects influence the performance of locally resonant PMMBs. To the best of our knowledge, this is the first study that systematically compares these three defect types in a single PMMB and demonstrates their respective contributions to voltage enhancement and integrity of the effective BG.

## 2. Structure and Performance Analysis for the Perfect PMMB

### 2.1. Structure of Perfect PMMB

Figure 1 shows the structural schematic of the perfect PMMB. The perfect PMMB can be considered as a 1 × 6 supercell. Each unit cell is composed of a lower aluminum substrate, two middle rubber plates with a centered piezoelectric plate (PZT-5H), and an upper cylindrical steel stub, which has the same central symmetry for the components. In each unit cell, the composite structure excluding the substrate is called the piezoelectric local resonator (PLR). In Figure 1a, *u*_1_ and *u*_2_ denote the excitation displacement and the corresponding response displacement of the PMMB in the z-direction, respectively. The six PLRs are named sequentially from left to right. The PLR nearest to the excitation source is referred to as the 1st PLR, while the farthest is termed the 6th PLR. In Figure 1b, *a* and *d* are the side length and the thickness of the aluminum substrate, respectively. *a*_1_ and *d*_1_ are the side length and the thickness of a rubber plate, respectively. The piezoelectric plate has the same side length as the rubber plate. *d*_2_ is the thickness of the piezoelectric plate. *r* and *h* are the radius and the height of the steel stub, respectively. The structural parameters of the unit cell are given as: *a* = 15 mm, *d* = 1 mm, *a*_1_ = 10 mm, *d*_1_ = 1 mm, *d*_2_ = 0.2 mm, *r* = 4.5 mm, and *h* = 10 mm. The material properties of the unit cell are given in Table 1. In Table 1, $\varepsilon_{ij}^{T}$ represents the permittivity at constant stress. The vacuum permittivity *ε*_0_ = 8.854 × 10^−12^ F/m.

### 2.2. Performance Analysis of the Perfect PMMB

A finite element model of the perfect PMMB is developed in COMSOL Multiphysics 6.0. For the band structure calculation, the dispersion curves are obtained via the supercell approach, in which the perfect PMMB is treated as an infinitely periodic structure without any external excitation. The Floquet-Bloch periodic boundary conditions are imposed on the boundaries of the unit cell, and the wave vector *k* is swept along the Γ–X direction within the first irreducible Brillouin zone, where Γ denotes the zone center, and X denotes the zone boundary along the beam length direction. This procedure allows the eigenfrequencies of the infinite periodic system to be extracted as a function of the wavenumber. To calculate the transmission spectrum, a finite beam with six unit cells is modeled with free-free boundaries at both ends to minimize end reflections. A transverse harmonic excitation displacement *u*_1_ is applied to the left end of the substrate, and a probe is used to measure the transverse response displacement *u*_2_. The transmission spectrum of the PMMB is defined as

(1) $$T=20\mathrm{lg}\left|\frac{u_{2}}{u_{1}}\right|,$$

where *T* is the transmission coefficient with the unit dB.

Figure 2a presents the dispersion curves along the Γ–X direction, from which a BG of 308–846 Hz is identified. Figure 2b shows the transmission spectrum of the perfect PMMB under a transverse harmonic excitation with amplitude *u*_1_ = 0.1 mm. The transmission coefficients fall below 0 dB over a comparable frequency range, yielding an effective attenuation band of 311–847 Hz that broadly agrees with the dispersion-curve values but is not exactly identical to them. The slight deviation is attributed to the different computational approaches: the BG comes from eigenfrequency analysis of an infinite periodic structure, while the attenuation band is from frequency–response simulations of the finite-sized structure. To distinguish the two bands, the following definitions are adopted: the BG derived from the dispersion curves is termed the theoretical BG (Figure 2a), and the attenuation band from the transmission spectrum is termed the effective BG (Figure 2b).

To explore the elastic wave attenuation mechanism inside the BG, we extracted four mode shapes from the dispersion curves at eigenfrequencies of 308 Hz, 478 Hz, 692 Hz, and 846 Hz, as shown in Figure 3. It can be seen from Figure 3a that at 308 Hz, there are higher z-directional vibration displacements at the PLRs than those at the aluminum substrate, which leads to most of the *z*-directional vibration energy being absorbed by the PLRs. Figure 3b and Figure 3c show *y*-directional in-plane shear vibration and the *x*-directional longitudinal shear vibration for the aluminum substrate at 478 Hz and 692 Hz, respectively. Thanks to the shear vibration mode of the PMMB, the z-directional propagation of the elastic wave is suppressed. It can be seen from Figure 3d that at 846 Hz, there are higher z-directional vibration displacements at the aluminum substrate than those at the PLRs, which leads to most of the *z*-directional vibration energy being absorbed by the aluminum substrate. At the two boundaries of the theoretical BG, the transverse propagation of the elastic wave is supported due to the curved vibration modes of the PMMB.

The open-circuit voltage outputs of the perfect PMMB are separately extracted from the six PLRs under a z-direction excitation displacement of *u*_1_ = 0.1 mm. Figure 4a–f show the voltage outputs for the six PLRs, respectively. It can be seen from Figure 4a–f that there are multiple voltage peaks in the low-frequency passband, along with an additional peak at the lower edge of the effective BG (311 Hz). At this frequency, the voltage outputs of the six PLRs are 15.28, 14.67, 12.78, 9.44, 5.01, and 1.29 V, respectively. Owing to the nearest distance from the excitation source, the 1st PLR shows the highest voltage output compared with other PLRs.

## 3. Effects of Structural Defects on PMMB Performance

The structural defects of the PMMB can be introduced by the variation of the natural frequencies of selected PLRs or the variation of the spacing between two adjacent PLRs. In this section, the effects of both mass defects and spacing defects on the vibration suppression and energy harvesting properties of the PMMB are studied.

### 3.1. Effects of Mass Defect in a Single PLR

Without altering the geometric dimensions of the PMMB, we introduce a mass defect solely in the 3rd PLR by setting its stub density to *ρ*_3_ = 2700 kg/m^3^, while keeping all other geometric and material parameters the same as those of the perfect PMMB configuration. The corresponding dispersion curves and transmission spectrum are presented in Figure 5a and Figure 5b, respectively. In Figure 5a, two nearly flat bands (376 Hz and 437 Hz) are observed within the theoretical BG, corresponding to the torsional mode and transverse mode of the 3rd PLR, respectively. Figure 5b shows a transmission peak at 437 Hz, which narrows the effective BG and consequently degrades the vibration suppression capability of the PMMB.

A parametric sweep of the stub density *ρ*_3_ of the 3rd PLR is performed over the range from 2000 kg/m^3^ to 10,000 kg/m^3^. The discrete values of 2000, 2700, 4000, 5000, 6000, 7000, 7800, 8960, and 10,000 kg/m^3^ are selected to examine the influence of stub density on the defect-mode frequencies and the corresponding open-circuit voltage outputs (see Figure 6). A non-uniform step size is deliberately adopted to include the densities of three representative engineering materials: aluminum (2700 kg/m^3^), steel (7800 kg/m^3^), and copper (8960 kg/m^3^), thus covering both lightweight and heavy structural materials of practical interest. Additional intermediate values are included to ensure a broad coverage of the density spectrum and to capture the overall trend of the performance variation. In Figure 6, the data point at *ρ*_3_ = 7800 kg/m^3^ corresponds to the perfect PMMB configuration without any defect. At this density, a voltage peak of 12.78 V is obtained at 311 Hz, which coincides with the lower edge of the effective BG. This point is plotted separately from the defected cases (*ρ*_3_ ≤ 7000 kg/m^3^ and *ρ*_3_ ≥ 8960 kg/m^3^) to clearly distinguish the reference baseline from the defected configurations. As shown in Figure 6, the defect-mode frequency exhibits a monotonic decrease with increasing *ρ*_3_. For *ρ*_3_ ≥ 7800 kg/m^3^, the frequency falls below the lower effective BG edge (311 Hz) and moves into the low-frequency passband, accompanied by a dramatic increase in voltage output. Because propagating waves in the passband continuously feed energy into the PLRs, the voltage outputs are consequently enhanced. Among all densities considered for lightweight design, *ρ*_3_ = 2700 kg/m^3^ offers the highest voltage output (23.56 V at 437 Hz) while maintaining a strongly localized defect state within the effective BG, making it a highly attractive candidate for practical applications.

To investigate the effect of stub density on the voltage outputs, Figure 7 presents the vibration shapes of the PMMB at the corresponding defect-mode frequencies. As shown in Figure 7a,b, at *ρ*_3_ = 2700 kg/m^3^ at its defect-mode frequency of 437 Hz, most of the energy is localized in the 3rd PLR (Figure 7a), where the vibration displacement is significantly higher than that in other PLRs and the substrate beam. As *ρ*_3_ = 5000 kg/m^3^, the phenomenon of energy localization in the 3rd PLR is weakened (Figure 7b), and some energy is dispersed to its adjacent PLRs. For *ρ*_3_ = 8960 and 10,000 kg/m^3^, which correspond to defect-mode frequencies of 294 Hz and 285 Hz, respectively, the vibration energy spreads across nearly all of the PLRs (Figure 7c,d) without localization at the defect. Nevertheless, the 3rd PLR exhibits relatively large displacements due to its occurrence in the passband, indicating that a higher voltage output is anticipated.

Figure 8 shows the open-circuit voltage outputs of the 3rd PLR with various mass defects. It can be seen from Figure 8a,b that as *ρ*_3_ = 2700 kg/m^3^, it has a significant energy location for the 3rd PLR at its defect-mode frequency of 437 Hz, at which it has a single peak voltage output of 23.56 V. As *ρ*_3_ = 5000 kg/m^3^, the low-frequency passband contains multiple voltage peaks, which consequently yields a reduced peak voltage of 0.82 V at the defect-mode frequency of 353 Hz. It can be seen from Figure 8c,d that as *ρ*_3_ = 8960 kg/m^3^, the peak voltage output of the 3rd PLR is 11.79 V at 294 Hz. As *ρ*_3_ = 10,000 kg/m^3^, the maximal voltage output of the 3rd PLR reaches 17.37 V at 285 Hz. It shows that in the low-frequency passband, with a lower defect-mode frequency of the PLR, it has a higher voltage output at this PLR. The results indicate that the energy localization mechanism within the effective BG is more effective in enhancing electrical output (23.56 V), despite the narrowing of the effective BG.

### 3.2. Effects of Mass Defects in Two PLRs

We also investigate the effects of the two mass defects on the vibration suppression and the energy harvesting performance of the PMMB. We use *ρ*_3_ = 5000 kg/m^3^ and *ρ*_5_ = 2700 kg/m^3^, and all other parameters are the same as those of the perfect PMMB. Figure 9a and Figure 9b present the dispersion curves and transmission spectrum, respectively, for the PMMB with mass defects at the 3rd and 5th PLRs. As shown in Figure 9a,b, three flat bands appear in the theoretical BG, corresponding to 353, 378, and 437 Hz. Among them, the mode shape of the PMMB at 378 Hz is the torsional mode of the 5th PLR, which has less effect on the transmission spectrum. Therefore, only two transmission peaks are observed above zero in the effective BG, and the emergence of these dual passbands reduces the effective BG width, compromising the vibration suppression performance of the PMMB.

Figure 10a and Figure 10b show the open-circuit voltage outputs from the 3rd and 5th PLRs for the PMMB with the two mass defects (*ρ*_3_ = 5000 kg/m^3^ and *ρ*_5_ = 2700 kg/m^3^), respectively. Figure 10a shows that the voltage outputs of the 3rd PLR at the defect-mode frequencies of 353 and 437 Hz are 4.94 V and 0.21 V, respectively. Compared with the single defect case (*ρ*_3_ = 5000 kg/m^3^, yielding 0.82 V at 353 Hz; Figure 8b), the voltage outputs are significantly improved under the dual mass defects. Figure 10b also shows voltage peaks at 353 and 437 Hz for the 5th PLR. However, owing to its greater distance from the excitation source, the 5th PLR yields a maximum voltage of only 0.79 V within the effective BG.

### 3.3. Effects of Spacing Defects Between Two Adjacent PLRs

According to the structure parameters of the unit cell given in Section 2.1, the center distance between adjacent PLRs in the perfect PMMB is 15 mm. A structural defect is introduced by increasing the length of the aluminum substrate and reducing the inter-PLR spacing through adjusting their relative positions, respectively. As the center distance between the two adjacent PLRs, *d_c_*, is not equal to 15 mm, it is considered that the structural defect is introduced in the PMMB.

#### 3.3.1. Effects of Increased Substrate Length Between Adjacent PLRs

In this section, a spacing defect is introduced by increasing the length of the aluminum substrate between the 3rd and 4th PLRs. Figure 11a–c show the defected structure, transmission spectrum, and vibration mode shape at 775 Hz for the PMMB with a spacing defect of *d_c_* = 30 mm between the 3rd and the 4th PLRs, respectively. In Figure 11a, the letter E points to the excited end of the PMMB, where the transverse excitation displacement (in the z-direction, Figure 1a) is given as *u*_1_ = 0.1 mm. It can be seen from Figure 11b that there is a transmission peak larger than zero at the defect frequency of 775 Hz in the effective BG. Owing to the band-pass effect of the defect-mode frequency, the width of the effective BG is reduced. It can be seen from Figure 11c that most of the energy is located at the position of the defective aluminum substrate, and it exhibits a curved vibration shape for the substrate beam at 775 Hz.

Figure 12a–c show the defected structure, transmission spectrum, and vibration mode shape for the PMMB with a spacing defect of *d_c_* = 45 mm between the 3rd and 4th PLRs, respectively, revealing a similar energy localization at the defective substrate position as observed in Figure 11a–c. The difference is that the defect frequency of the PMMB is reduced from 775 Hz to 661 Hz, which leads to an increase in the maximal vibration displacement of the substrate beam from 0.122 mm to 0.354 mm.

The above analysis indicates that when a spacing defect is introduced by increasing the substrate length between two adjacent PLRs, most of the vibration energy becomes localized at the defected position of the substrate beam. From an energy harvesting perspective, the optimal approach is to attach a piezoelectric transducer to the defected substrate beam rather than to the PLRs. A piezoelectric plate with the size of 10 mm × 10 mm × 0.2 mm is pasted to the defected position of the substrate beam. Figure 13 shows the voltage outputs of the piezoelectric substrate beam with two different spacing defects. Figure 13a,b show the voltage outputs of the piezoelectric substrate beam with two different spacing defects. It can be seen from Figure 13a and Figure 13b that the open-circuit voltage outputs of the piezoelectric plate located at the substrate beam are 9 V at 767 Hz for the configuration of *d_c_* = 30 mm and 25 V at 655 Hz for the configuration of *d_c_* = 45 mm in the effective BG, respectively. The results show that the energy harvesting capacity of the substrate beam can be improved by an increase in the defect length of the substrate beam. It is noted that since a small-sized piezoelectric plate is added to the substrate beam, the defect-mode frequencies of the PMMB in this analysis are slightly different from the values of 775 Hz and 661 Hz obtained for the configurations without this additional plate (see Figure 11 and Figure 12).

#### 3.3.2. Effects from Reduced Spacing Between Two Adjacent PLRs

In this section, the spacing defect is introduced by reducing the center-to-center distance for a pair of adjacent PLRs, while keeping the substrate beam length unchanged and only shifting the PLR position along the beam. The 1st and 2nd PLRs are moved in opposite directions until the center distance *d_c_* reaches 12 mm (Figure 14a). Figure 14b and Figure 14c present the transmission spectrum and the mode shape at 301 Hz for the PMMB with this spacing defect, respectively. As can be seen from Figure 14b, the effective BG width is not affected by the defect compared with that of the perfect PMMB (Figure 2b). The difference is that an extremely high transmission coefficient appears at 301 Hz in the low-frequency passband. It is observed from Figure 14c that both the 3rd and 5th PLRs exhibit significantly higher displacements than the remaining PLRs in the PMMB, indicating that these two resonators possess a strong energy harvesting potential at 301 Hz.

In order to study the effect of the spacing reduction between the 1st and 2nd PLRs on the energy harvesting performance of the PMMB, we compare the maximal open-circuit voltage outputs of the six PLRs for various defect spacings between the 1st and 2nd PLRs. Figure 15 shows that reducing the spacing between the 1st and 2nd PLRs to *d_c_* = 12 mm yields extremely high voltage peaks for the 1st, 2nd, 3rd, 5th, and 6th PLRs, while further reduction to *d_c_* = 11 mm only enhances the peak from the 4th PLR (relative to *d_c_* = 12 mm) but sharply reduces those of the others, indicating the existence of an optimal spacing for maximizing the peak voltage outputs. In Figure 15, the maximal voltage outputs for the configuration of *d_c_* = 12 mm are collected at 301 Hz. For the other configurations, the maximal voltage outputs are observed either at the lower effective BG edge (311 Hz) or in the adjacent low-frequency passband.

The effects of the reduced spacings between the 2nd–3rd and the 3rd–4th PLR pairs are also studied. Figure 16a,b show the maximum open-circuit voltage outputs of the six PLRs for varying spacings between the two pairs. The results reveal that reducing the spacing to *d_c_* = 12 mm yields relatively higher voltage peaks for all six PLRs. Compared with the optimal outputs from the 1st–2nd PLR pair (Figure 15) and the 2nd–3rd PLR pair (Figure 16a), the 3rd–4th PLR pair (Figure 16b) yields lower open-circuit voltages, suggesting that adjusting the spacing between adjacent PLRs near the excitation source effectively enhances the system’s energy harvesting capability.

To quantify the global effect of the position-change spacing defect on energy distribution, Table 2 compares the average voltages of the PLRs adjacent to the defect and those farthest from it, together with the ratio of these two voltages, the lower BG edge, and the summed voltage. The adjacent PLR group is defined as the two PLRs that are physically displaced (i.e., the two PLRs that were moved toward each other to introduce the defect), while the farthest PLR group refers to the two PLRs located furthest from the displaced region. The voltage ratio between the adjacent and farthest PLRs serves as a quantitative indicator of the underlying physical mechanism: a ratio greater than unity implies energy localization near the displaced resonators, whereas a ratio less than unity indicates that energy is redirected toward the distal end. The summed voltage, calculated as the arithmetic sum of all six PLR voltages at the major peak frequency, serves as a theoretical indicator of global energy output.

As shown in Table 2, moving two PLRs does not alter the effective BG for all cases, but it significantly redistributes energy. The 1st–2nd spacing defect yields the highest summed voltage (775.8 V, more than 13 times that of the perfect PMMB) and shifts the major peak frequency from 311 Hz (Lower edge of effective BG) to 301 Hz. This suggests that a properly designed position-change spacing defect achieves high voltage primarily through defect-resonance-enhanced propagating-wave energy transport, rather than through purely localized standing-wave resonance.

The adjacent-to-farthest PLR voltage ratios in Table 2 are below unity for 1st–2nd and 2nd–3rd spacing defects, confirming that moving two PLRs toward each other redirects energy toward the distal end. In contrast, the 3rd–4th spacing defect yields a ratio greater than unity (1.81). However, this does not imply strong localization; rather, it arises because the 6th PLR falls outside the interference region, which produces very low voltage (2.29 V, Figure 16b). The total voltage generated by the six PLRS due to the 3rd–4th spacing defect (92.6 V) is significantly lower than that obtained by the 1st–2nd and 2nd–3rd spacing defects (775.8 V and 732.8 V, respectively), suggesting that the centrally located defect is relatively inefficient for maximizing global energy output.

## 4. Effects of Electrical Defects on PMMB Performance

An electrical defect can be introduced by connecting an external inductor to the piezoelectric plate of a PLR while leaving the electrical conditions of the other PLRs unchanged. The electrical resonance frequency in an inductor-capacitor circuit can be expressed as

(2) $$f=\frac{1}{2\pi \sqrt{LC_{p}}},$$

where *L* is the external inductance, and *C_p_* is the capacitance of the piezoelectric plate in a PLR. The capacitance is expressed as

(3) $$C_{p}=\frac{\varepsilon_{33}^{T}a_{1}a_{1}}{d_{2}},$$

where $\varepsilon_{33}^{T}$ is the dielectric constant of the piezoelectric plate. *a*_1_ and *d*_2_ are the side length and thickness of the piezoelectric plate, respectively. In the following analysis, the parameters are given as: *a*_1_ = 10 mm, *d*_2_ = 0.2 mm, $\varepsilon_{33}^{T}$ = 3400*ɛ*_0_.

### 4.1. Effect of Electrical Defect Induced by an Additional Inductor

An external inductor of *L* = 12 H is connected to the 3rd PLR. According to Equations (2) and (3), the electrical resonant frequency is calculated as *f* = 375 Hz. Figure 17a and Figure 17b show the dispersion curves and the transmission spectrum for the PMMB with the electrical defect of the 3rd PLR, respectively. It can be seen from Figure 17a that there are two flat bands in the theoretical BG. Among them, the flat band at 375 Hz corresponds to the electrical resonance frequency of the inductor-capacitor circuit. Figure 17b shows no displacement peaks greater than zero within the effective BG, confirming that vibration suppression remains perfect. Figure 18 reveals two relatively higher voltage peaks (28 V and 3.5 V), corresponding to the lower effective BG edge (311 Hz) and the electrical resonance frequency (375 Hz), respectively. The peak at 375 Hz (3.5 V) exhibits negligible transmission (Figure 17b) and therefore does not contribute to actual energy harvesting. Although the 375 Hz voltage peak is unrelated to harvested energy, its presence confirms that electrical resonance can produce voltage independently of mechanical displacement. The absence of a transmission peak at this frequency verifies that vibration suppression remains uncompromised.

### 4.2. Effect of Electrical Defect Induced by Two Additional Inductors

To explore frequency tunability of the electrical response, we introduce two electrical defects by connecting external inductors of 12 H and 8 H to the 3rd and 5th PLRs, respectively. Figure 19a and Figure 19b show the dispersion curves and the transmission spectrum for the PMMB with the two electrical defects, respectively. There are three flat bands in the effective BG, two of which correspond to the electrical resonance frequencies of the LC circuits at 375 and 458 Hz, with no corresponding transmission peaks above zero. In contrast to the perfect PMMB (Figure 2b) and the single-electrical-defect case (Figure 17b), the dual-inductor configuration yields a remarkably high transmission coefficient at 269 Hz in the low-frequency passband. Figure 20a and Figure 20b show the open-circuit voltage outputs for the 3rd and 5th PLRs under the two electrical defects, respectively. It can be seen from Figure 20a,b that there are extremely high voltage peaks occurring at 269 Hz for both the 3rd and the 5th PLRs. In addition, voltage peaks are observed at the electrical resonance frequencies of the shunted PLRs, namely 3.7 V at 375 Hz for the 3rd PLR and 1.9 V at 458 Hz for the 5th PLR. These voltage responses arise from the LC resonance of the shunt circuit rather than from mechanical strain, as indicated by the negligible change in the transmission spectrum (Figure 19), and therefore do not constitute mechanical-to-electrical energy conversion. With the dual inductors, the voltage peak at the original effective BG edge (311 Hz) is significantly reduced, while a pronounced peak emerges at a lower frequency (269 Hz) in the passband. This indicates that the electrical defects effectively redistribute the electromechanical response toward lower frequencies, thereby improving the low-frequency energy harvesting capability.

## 5. Discussion

The comparison in Table 3 allows a systematic examination of the five defect configurations from three key perspectives: effective BG integrity, voltage output characteristics, and their underlying physical mechanisms. These comparisons help to identify the trade-offs associated with different defect types and offer useful guidance for defect selection in dual-functional PMMBs.

As shown in Table 3, either the stub-density-reduced mass defect or the substrate-length-increased spacing defect generates defect states inside the effective BG, with peak voltages of 23.56 V and 25 V, respectively. However, both configurations narrow the effective BG due to the transmission peaks introduced within it, which somewhat reduces the vibration suppression performance. The mass defect harvests voltage from the defected PLR itself, while the substrate-length-increased spacing defect harvests voltage from the PZT patch attached to the deflected beam between the 3rd–4th PLRs. Notably, the stub-density-reduced mass defect (*ρ*_3_ = 2700 kg/m^3^) requires no geometric modification and offers a lightweight advantage over the substrate-length-increased spacing defect.

By contrast, the position-change spacing defect leaves the BG unchanged. The position-change spacing defect (*d_c_* = 12 mm) achieves global energy redistribution through defect–boundary interference (i.e., interference between the defect-scattered and boundary-reflected waves), yielding the highest summed voltage of 775.8 V across all six PLRs, more than 13 times that of the perfect PMMB. This output is spatially distributed rather than concentrated at a single resonator.

The electrical defects also maintain the effective BG, but their output characteristics are different. The voltage is harvested from the shunted PLRs. The single-inductor configuration delivers a primary peak of 28 V at the lower effective BG edge, while the dual-inductor configuration shifts the major response to the passband. These major responses are accompanied by finite transmission and thus represent genuine mechanical-to-electrical energy conversion. Notably, for electrical defects, an additional minor voltage peak appears at the pure electrical resonance frequency inside the effective BG, but it does not constitute energy harvesting due to negligible transmission.

The physical mechanisms underlying these configurations highlight their fundamental distinctions. Stub-density-reduced mass defect and the substrate length- increased spacing defect both create defect states via classical localization, by altering either the local mass or the substrate stiffness, respectively. The position-change spacing defect primarily redirects energy toward the distal end through defect–boundary interference. The electrical defects, meanwhile, generate voltage responses through LC resonance, with the major peak appearing either at the lower effective BG edge or in the passband depending on the specific inductor configuration.

These trends are qualitatively consistent with previous studies. Mass- or stiffness-induced defect states in PCs and locally resonant metamaterials have been widely reported to create localized modes inside the BG and induce transmission peaks [22,23]. The energy redistribution induced by the position-change spacing defect is consistent with boundary reflections and scattering observed in finite-size metamaterials [24] and with defect-mode-enabled energy localization reported in PCs [25]. Together, these observations suggest a defect–boundary interference mechanism in the present work. Electrically controlled defects have also been explored for multi-band wave localization in PMMs [26]. However, previous studies have mainly focused on defect-mode tunability or wave localization, rather than on vibration suppression and energy harvesting for dual-functional PMMBs. In addition, they rarely compare different defect types within the same structure. The present work therefore goes beyond these studies by systematically comparing mass, spacing, and electrical defects on the same PMMB structure, revealing their distinct roles in dual-functional PMMBs.

It should be noted, however, that the present work focuses on representative single- and dual-defect configurations to isolate individual defect mechanisms. In practical engineering structures where multiple defects coexist, their mutual coupling could introduce additional defect states and more complex energy redistribution, potentially affecting the effective BG and harvesting performance. Such multi-defect scenarios are not considered in the present study, as the objective is to establish a systematic comparison of individual defect types rather than to model complex defect ensembles.

In summary, among the defect types that preserve the BG, the PLR position-shift-induced spacing defect maximizes global output through energy redistribution. The electrical defects provide frequency-flexible voltage enhancement at the effective BG edge or in the passband, without compromising vibration suppression. In contrast, the stub mass defect or substrate-length-change spacing defects deliver substantial voltage outputs by generating defect states inside the effective BG, albeit at the cost of narrowed effective BGs. These findings provide a mechanistic understanding of defect-type selection in dual-functional PMMBs.

## 6. Conclusions

This paper systematically investigates the effects of structural and electrical defects on the vibration suppression and energy harvesting performance of a locally resonant PMMB. Three types of defects are considered: mass defects of the PLRs, spacing defects of adjacent PLRs, and electrical defects realized by connecting inductors to selected PLRs. For the considered defect configurations, the main conclusions are as follows:

- The stub-density-change-induced mass defect and substrate-length-change-induced spacing defect generate localized states via modification of local mass and stiffness, respectively, yielding high voltage while narrowing effective BG. From a lightweight design perspective, *ρ*_3_ = 2700 kg/m^3^ (corresponding to aluminum) is identified as the optimal value, owing to its excellent energy localization on a single resonator and the resulting maximum voltage output.
- The PLR-position-change-induced spacing defect (*d_c_* = 12 mm) preserves the effective BG while yielding the highest total energy output across all six PLRs, through energy redistribution induced by defect–boundary interference. Depending on the spacing value, the enhanced response can occur either at the lower effective BG edge or in the passband, which appears to differ from classical defect-state localization.
- The single-inductor and dual-inductor configurations generate voltage responses through LC resonance, with peaks at the lower effective BG edge and in the passband, respectively. Minor voltage peaks are also observed at pure electrical resonance frequencies, yet they are ineffective for energy harvesting due to negligible transmission within the effective BG.

Based on the present results, the position-change spacing defect is most effective for maximizing global energy output; the single-inductor electrical defect is well-suited for localized voltage enhancement without compromising vibration suppression; the dual-inductor electrical defect provides a practical solution for low-frequency applications; and density-reduced mass defect or substrate-length-increased spacing defect generate defect states inside the effective BG, at the cost of narrowing it. Compared with previous studies that typically focus on a single defect type, this work provides a systematic comparison of these distinct mechanisms, offering guidance for defect-type selection in dual-functional PMMBs. Several directions remain for future investigation, including random excitation, load power testing, multi-defect coupling, and alternative low-inductance circuit schemes. Although beyond the scope of this work, these aspects could further advance the practical application of defect-configured PMMBs.

## Figures and Tables

**Figure 1:**
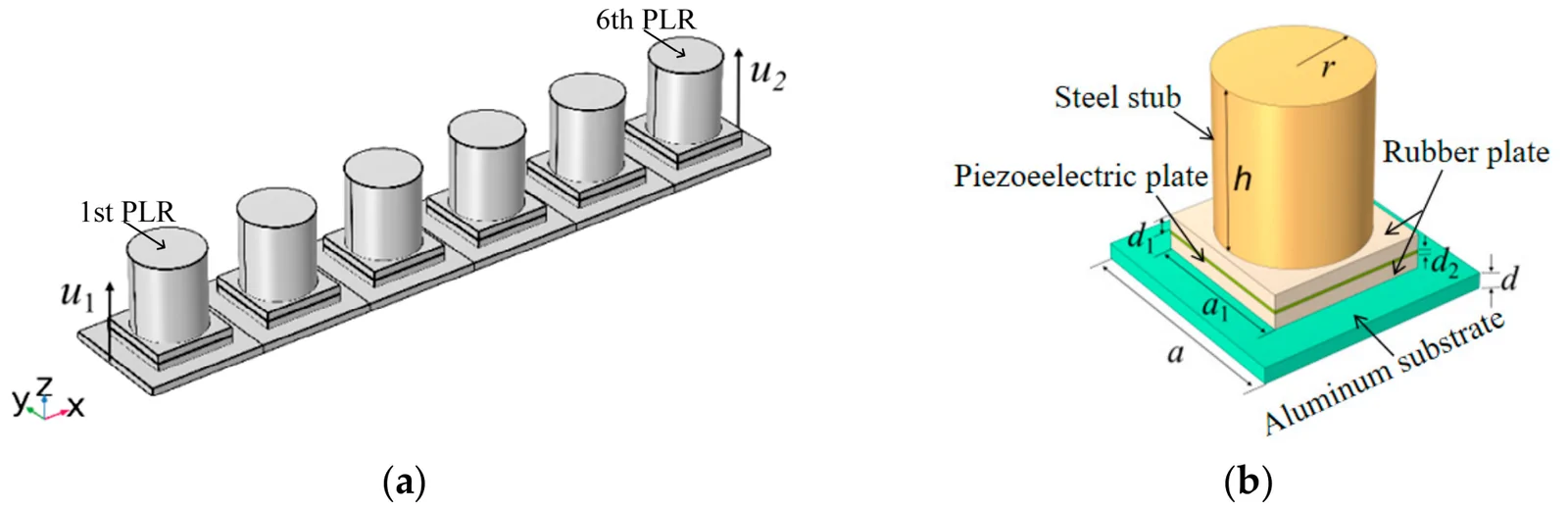
Structural schematic of the perfect PMMB: (**a**) supercell; (**b**) unit cell.

**Figure 2:**
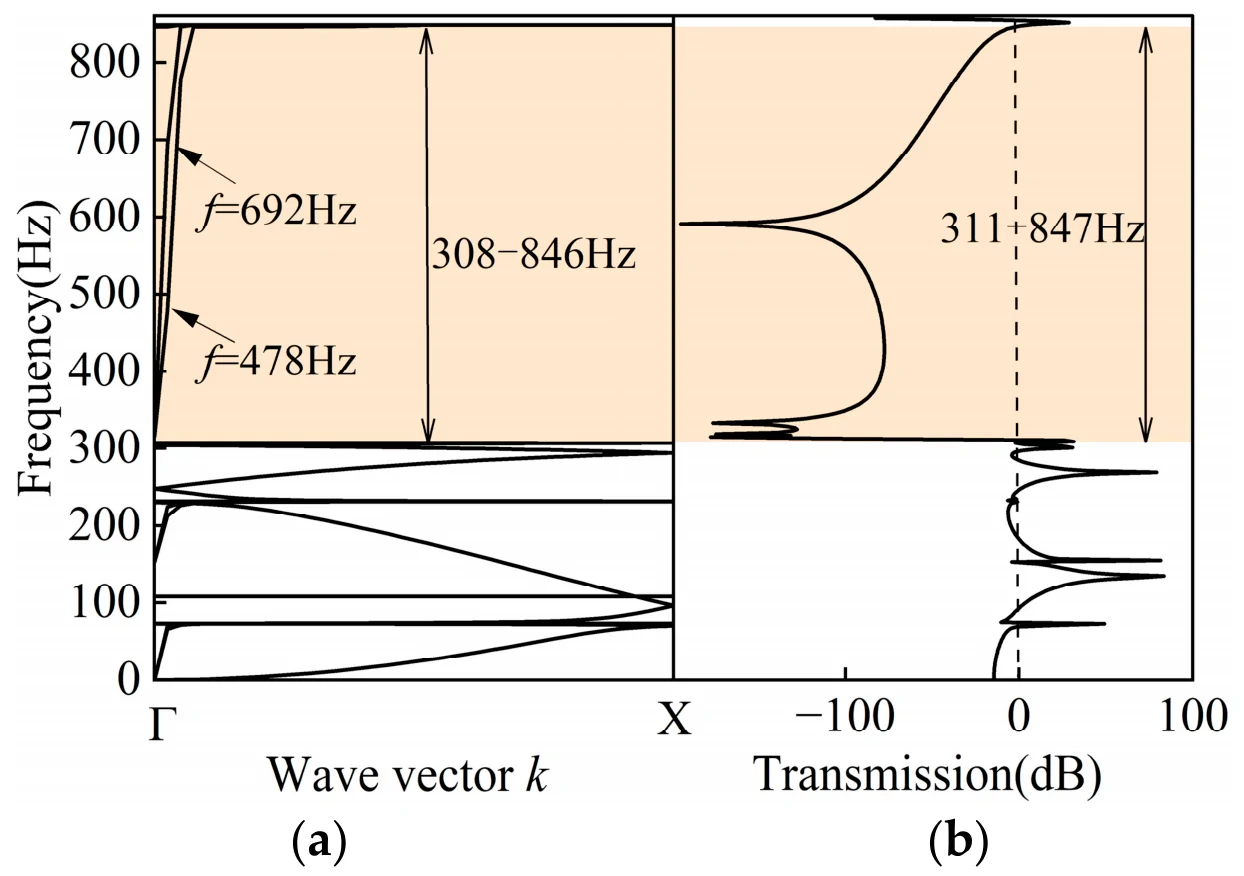
(**a**) Dispersion curves and (**b**) transmission spectrum for the perfect PMMB.

**Figure 3:**
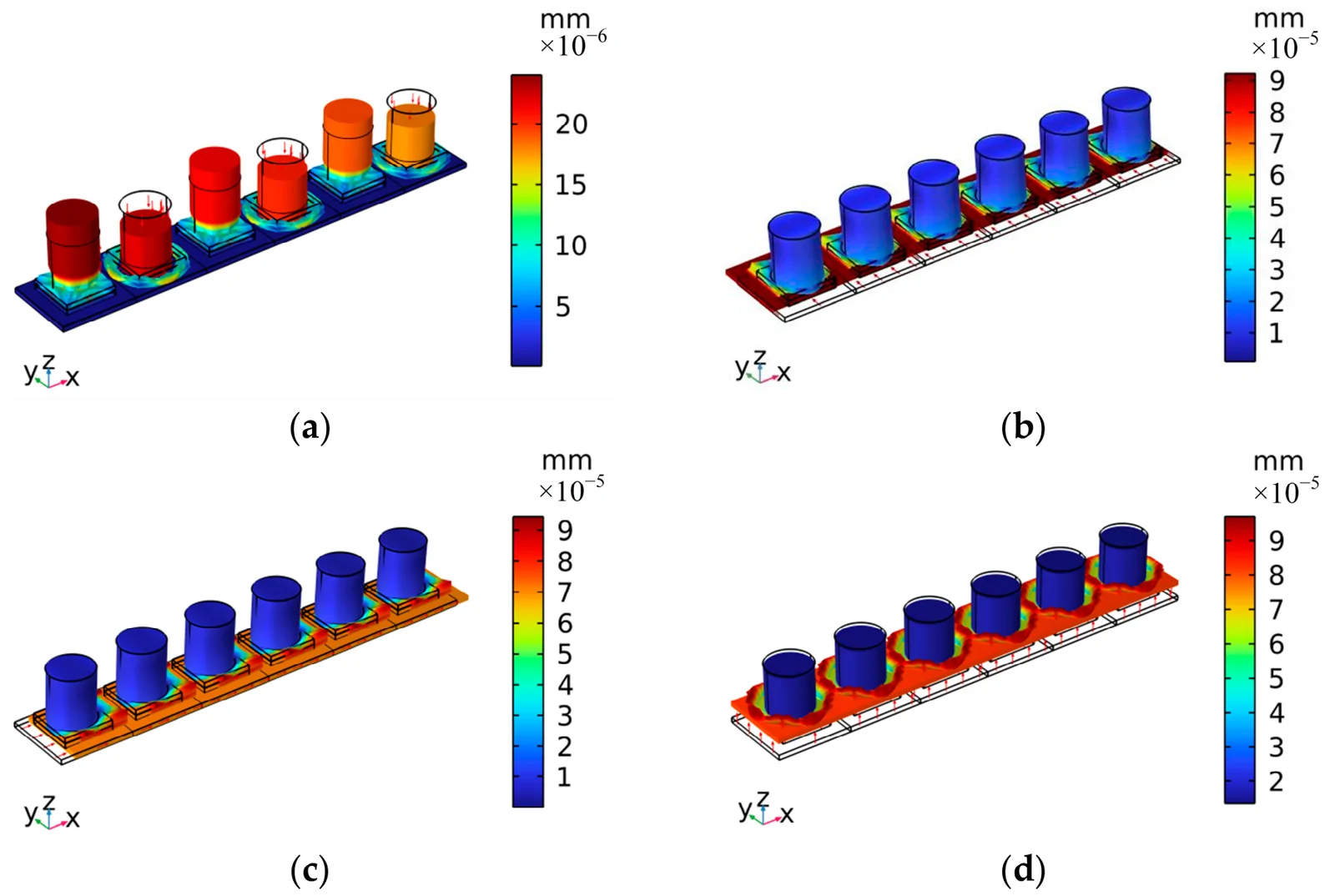
Mode shapes of the perfect PMMB at selected eigenfrequencies within the theoretical BG: (**a**) 308 Hz; (**b**) 478 Hz; (**c**) 692 Hz; (**d**) 846 Hz (unexcited).

**Figure 4:**
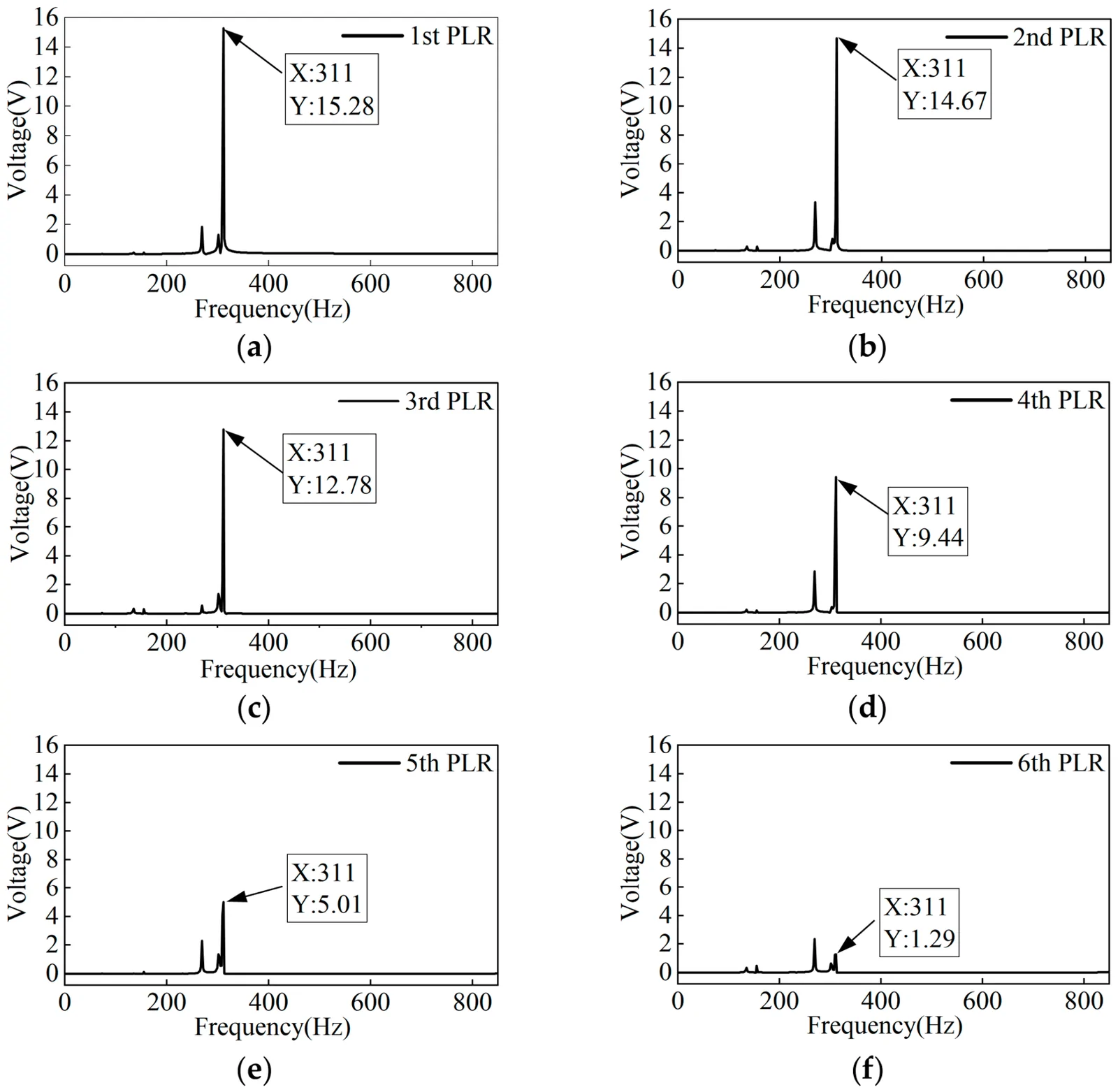
Open-circuit voltage outputs for six PLRs in perfect PMMB: (**a**) 1st PLR; (**b**) 2nd PLR; (**c**) 3rd PLR; (**d**) 4th PLR; (**e**) 5th PLR; (**f**) 6th PLR. Excitation: *u*_1_ = 0.1 mm.

**Figure 5:**
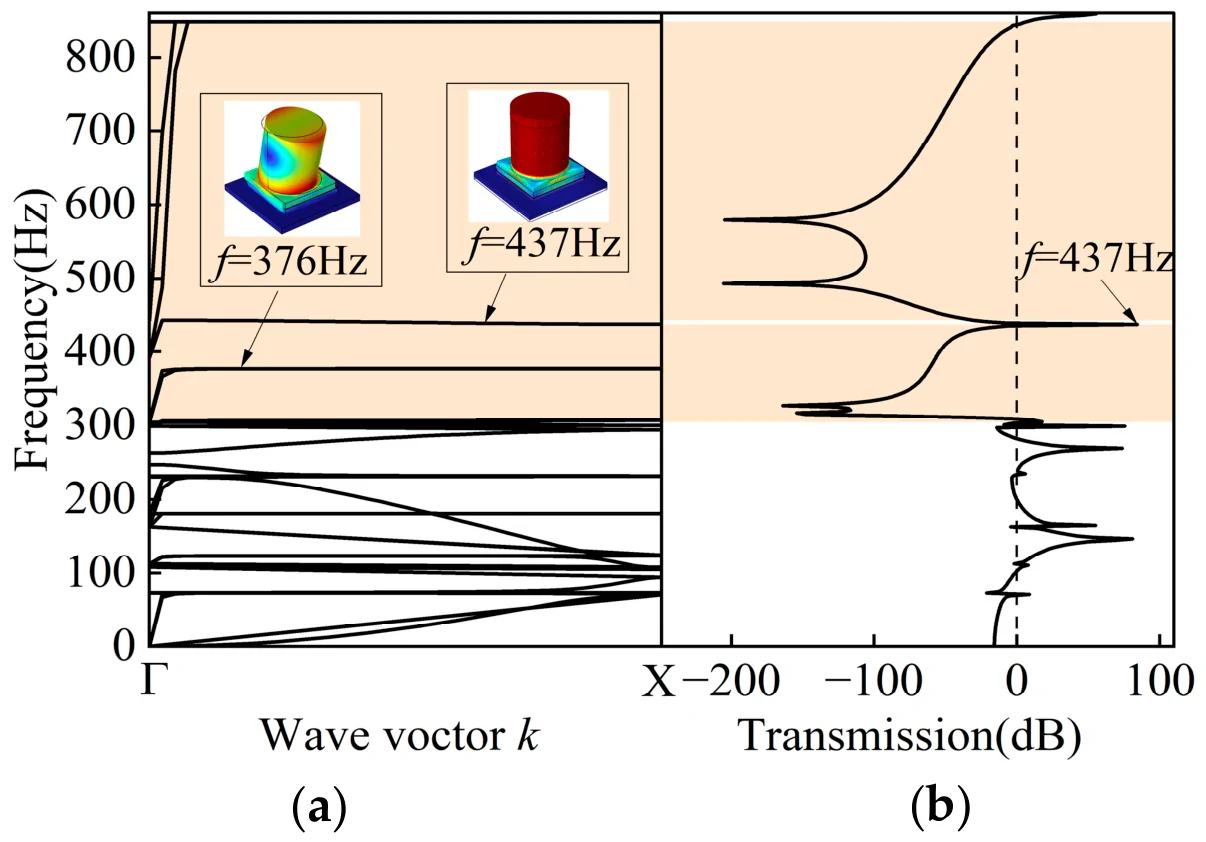
(**a**) Dispersion curves and (**b**) transmission spectrum for a PMMB with mass defect from the 3rd PLR (*ρ*_3_ = 2700 kg/m^3^).

**Figure 6:**
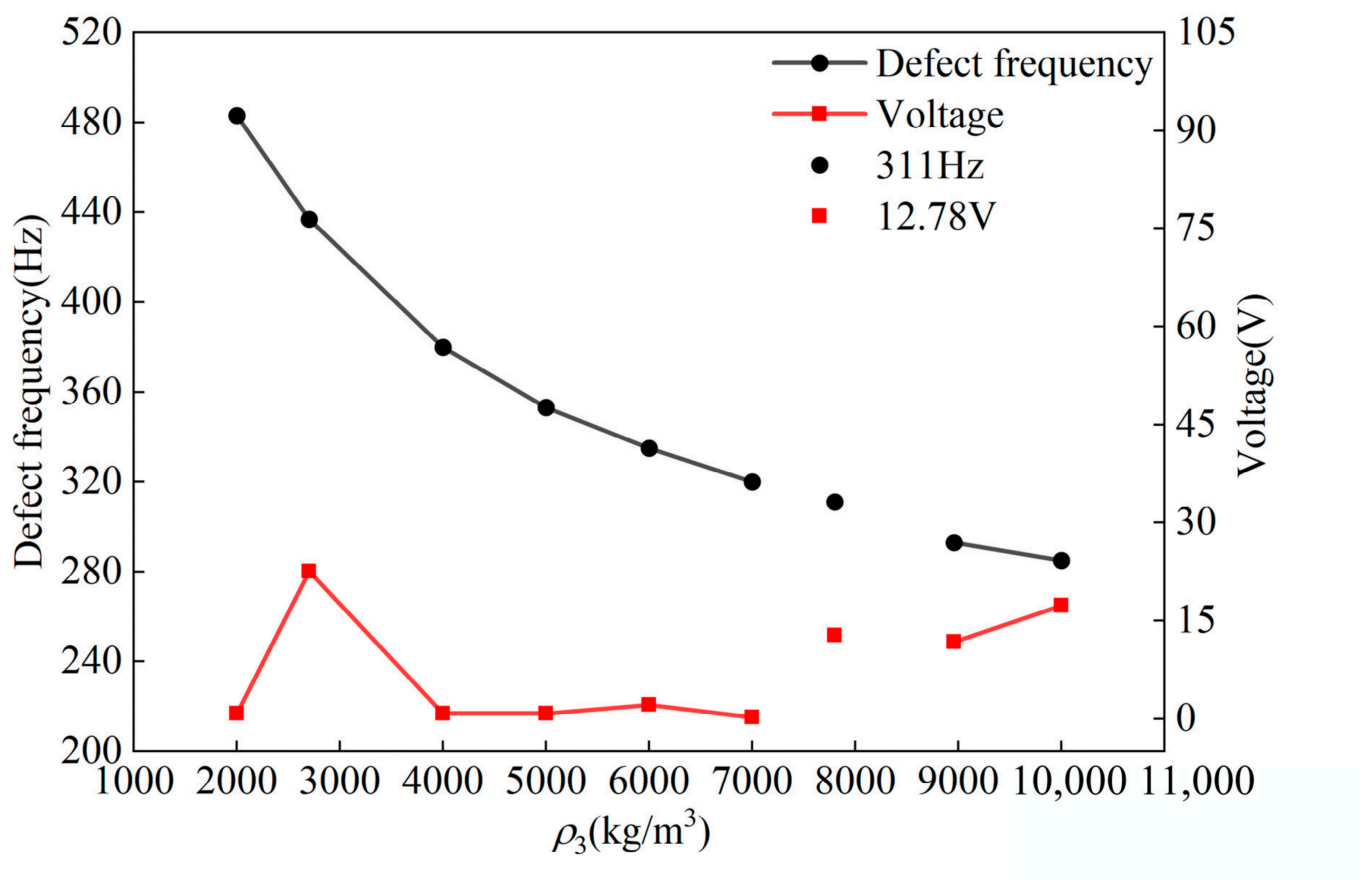
Variation of defect-mode frequency and open-circuit voltage with stub density in the 3rd PLR. The 7800 kg/m^3^ point (defect-free reference) is isolated for clarity. Black circle: BG lower edge (311 Hz); red square: perfect PMMB output (12.78 V). Excitation: *u*_1_ = 0.1 mm.

**Figure 7:**
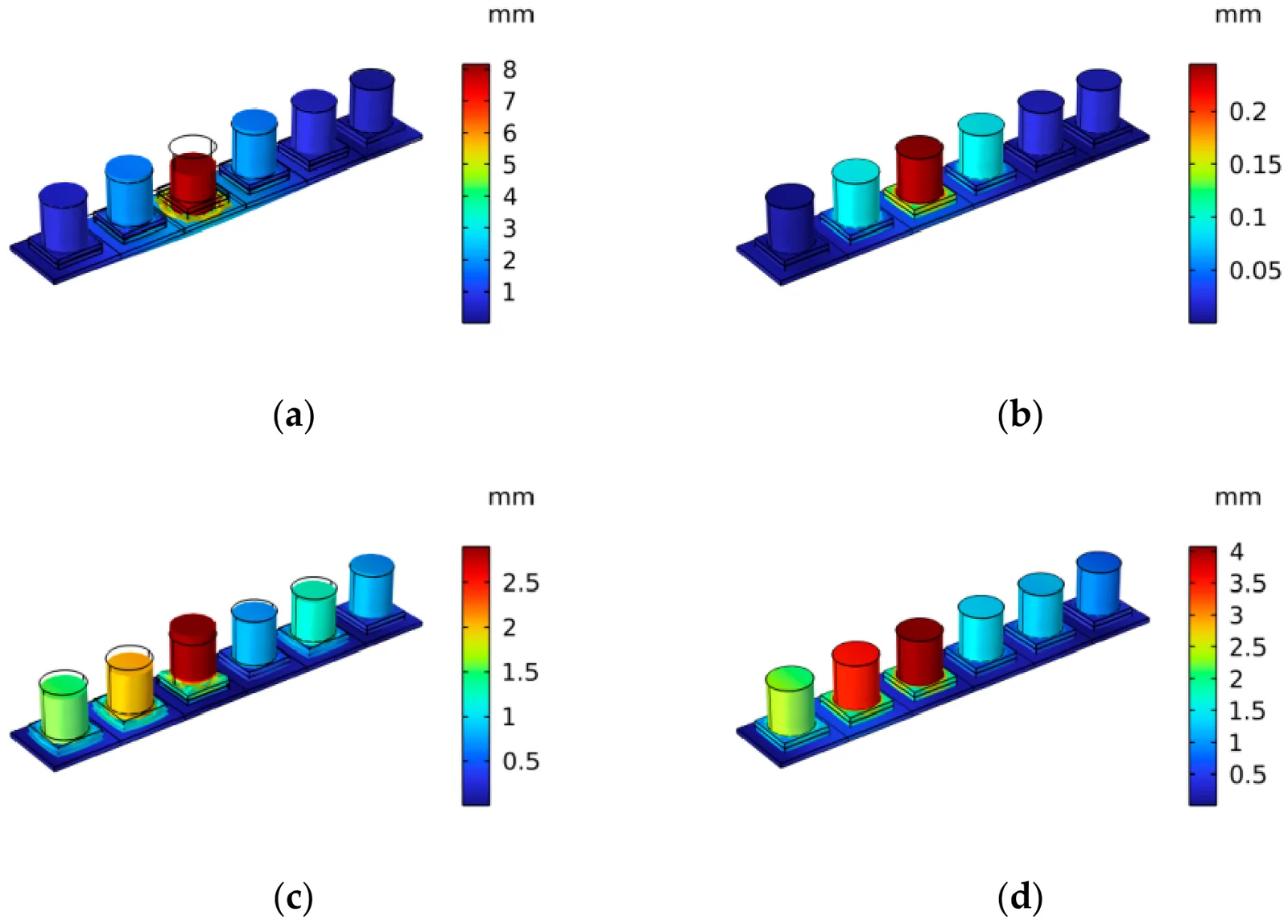
Vibration mode shapes at defect-mode frequencies for the 3rd PLR mass defects: (**a**) *ρ*_3_ = 2700 kg/m^3^ (437 Hz); (**b**) *ρ*_3_ = 5000 kg/m^3^ (353 Hz); (**c**) *ρ*_3_ = 8960 kg/m^3^ (294 Hz); (**d**) *ρ*_3_ = 10,000 kg/m^3^ (285 Hz). Excitation: *u*_1_ = 0.1 mm.

**Figure 8:**
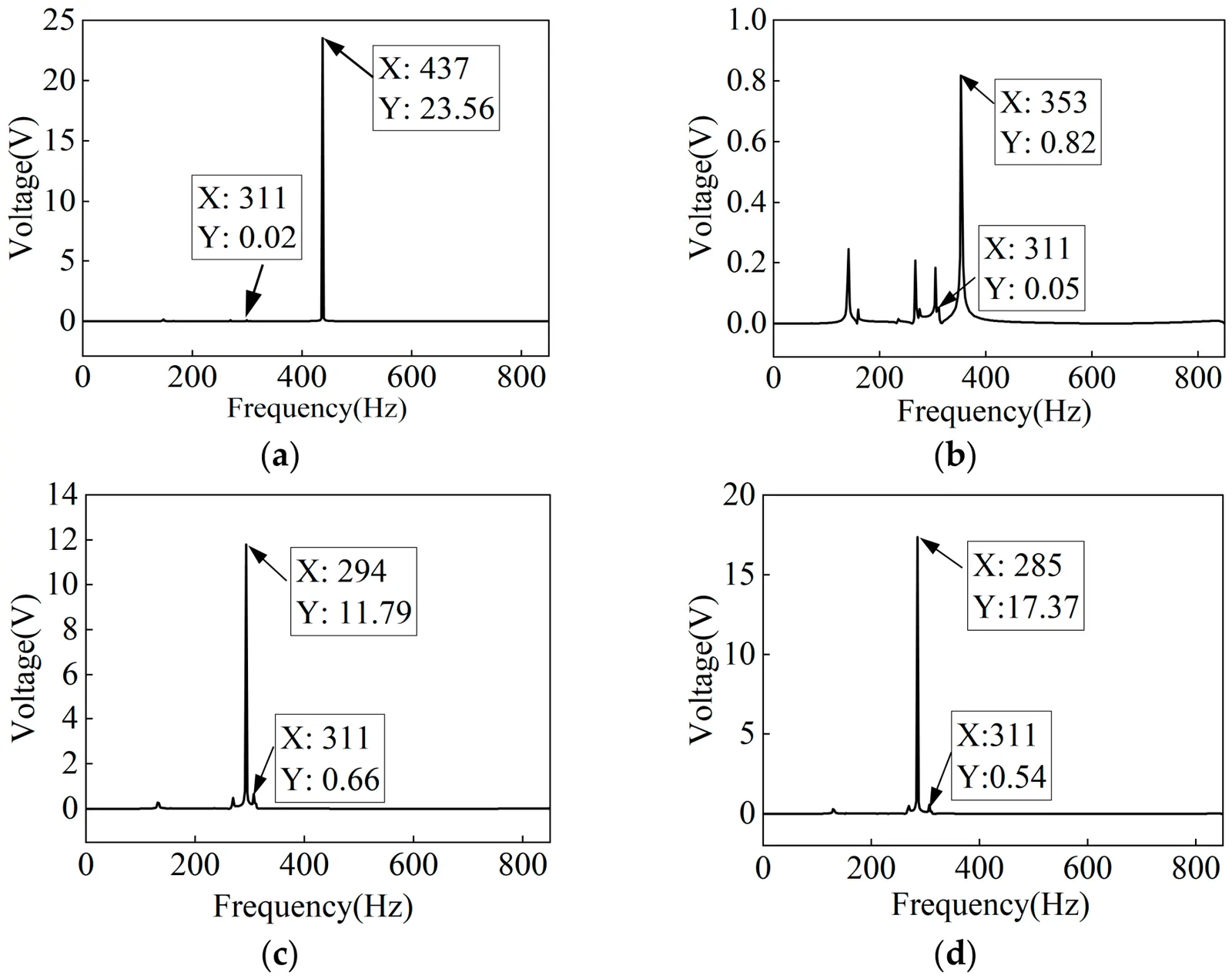
Open-circuit voltage outputs of the 3rd PLR for a PMMB with different mass defects (varying stub density of this PLR): (**a**) *ρ*_3_ = 2700 kg/m^3^; (**b**) *ρ*_3_ = 5000 kg/m^3^; (**c**) *ρ*_3_ = 8960 kg/m^3^; (**d**) *ρ*_3_ = 10,000 kg/m^3^. Excitation: *u*_1_ = 0.1 mm.

**Figure 9:**
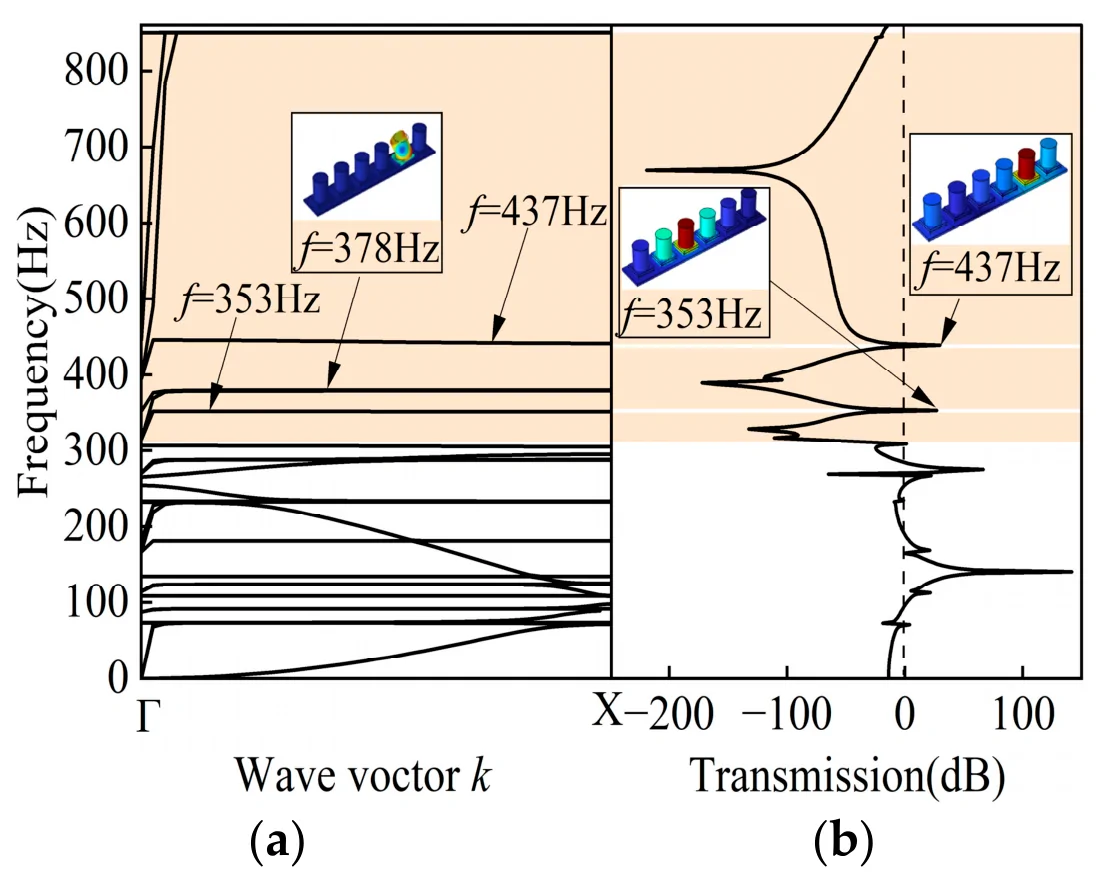
(**a**) Dispersion curves and (**b**) transmission spectrum for a PMMB with mass defects in the 3rd and the 5th PLRs (*ρ*_3_ = 5000 kg/m^3^, *ρ*_5_ = 2700 kg/m^3^).

**Figure 10:**
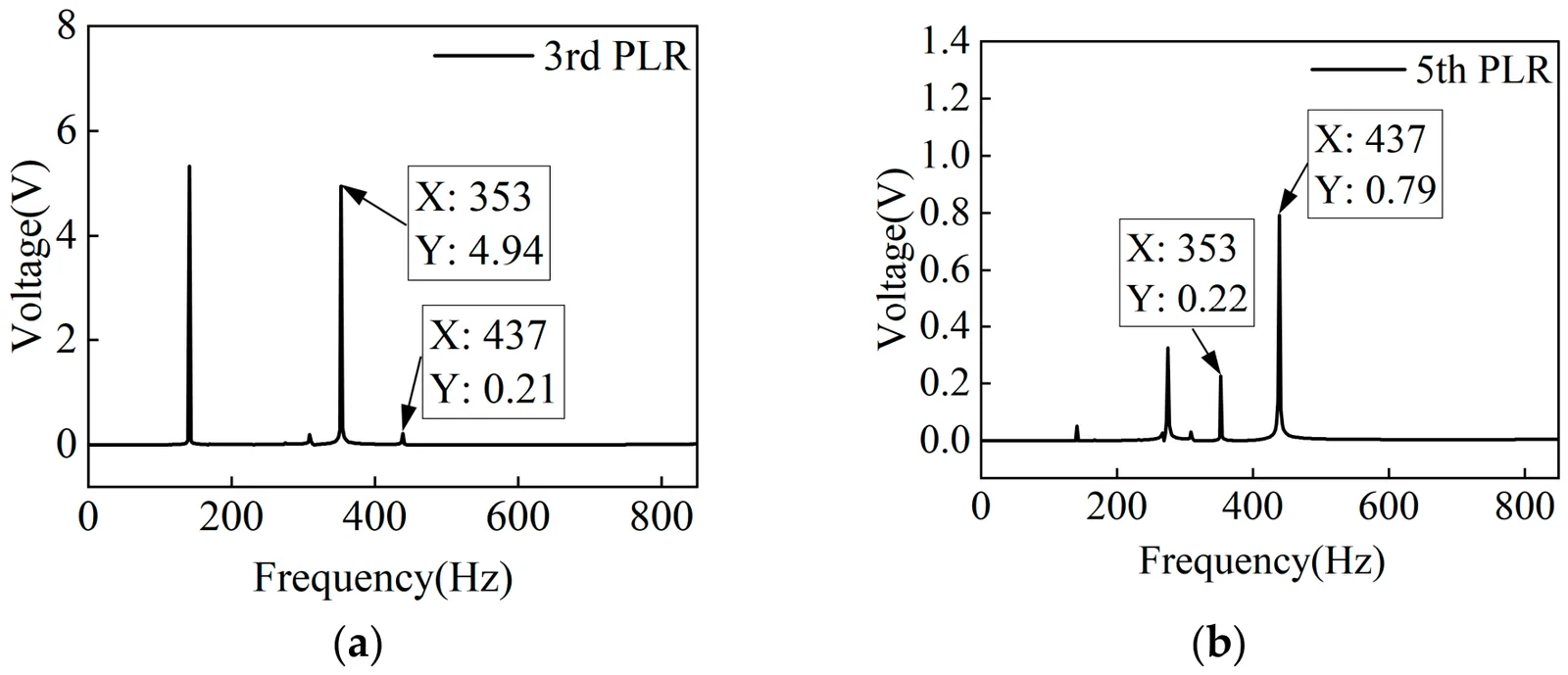
Open-circuit voltage outputs of the 3rd and 5th PLRs of the PMMB with dual mass defects (*ρ*_3_ = 5000 kg/m^3^, *ρ*_5_ = 2700 kg/m^3^): (**a**) 3rd PLR; (**b**) 5th PLR. Excitation: *u*_1_ = 0.1 mm.

**Figure 11:**
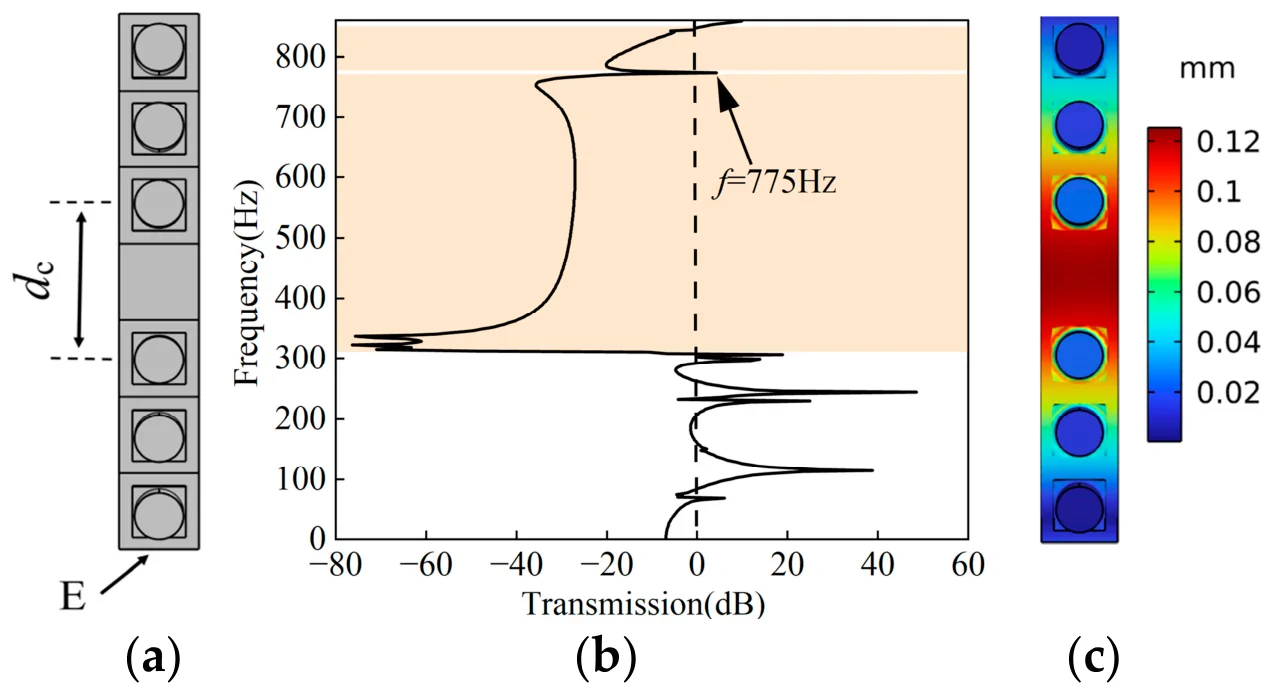
PMMB with a spacing defect of *d_c_* = 30 mm between the 3rd and 4th PLRs: (**a**) top view of defective PMMB; (**b**) transmission spectrum; (**c**) top view of vibration mode shape at defect-mode frequency (775 Hz). Excitation: *u*_1_ = 0.1 mm.

**Figure 12:**
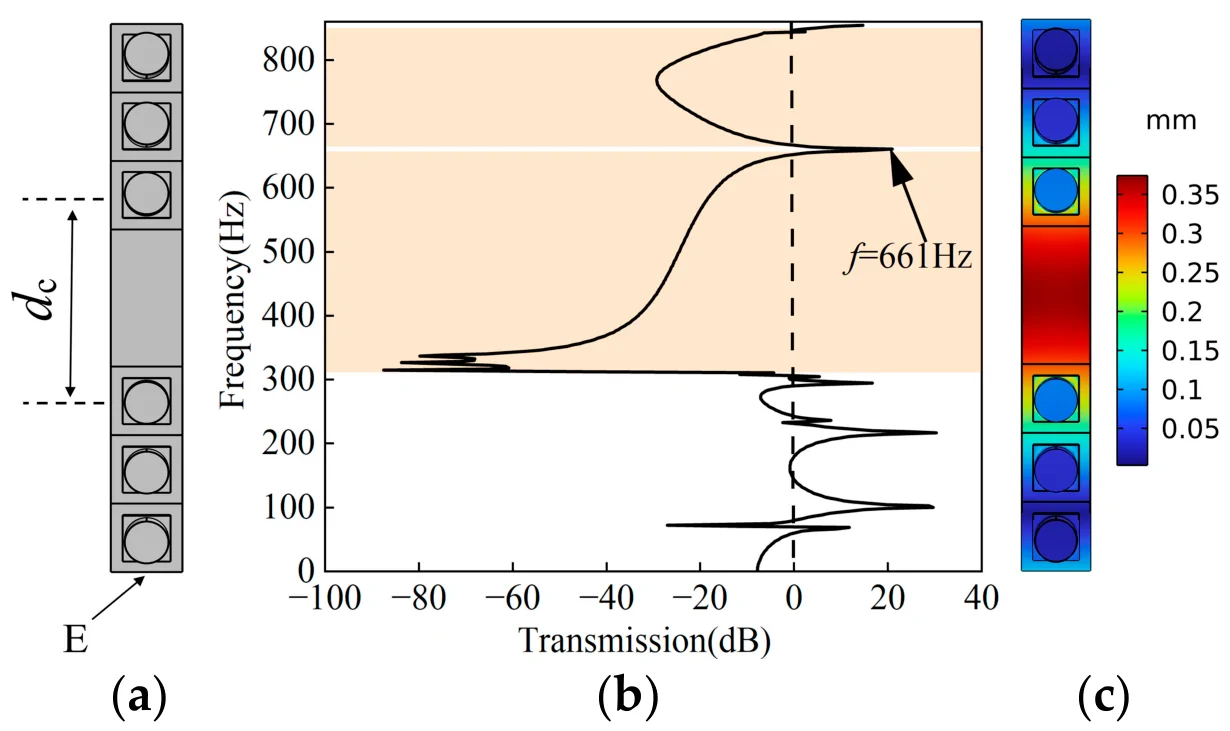
PMMB with a spacing defect of *d_c_* = 45 mm between the 3rd and 4th PLRs: (**a**) top view; (**b**) transmission spectrum; (**c**) top view of vibration mode shape at defect-mode frequency (661 Hz). Excitation: *u*_1_ = 0.1 mm.

**Figure 13:**
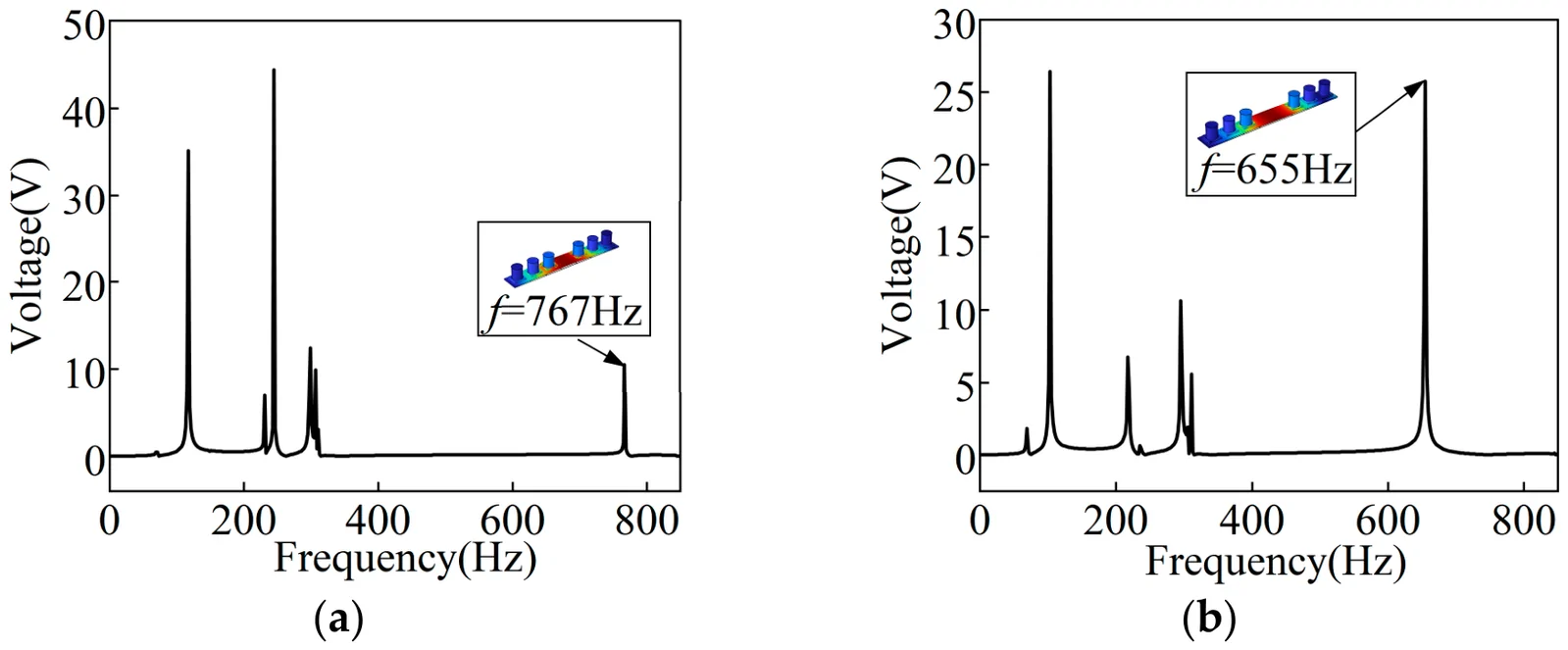
Open-circuit voltage outputs of the substrate beam in PMMB with different spacing defects: (**a**) *d_c_* = 30 mm; (**b**) *d_c_* = 45 mm. Excitation: *u*_1_ = 0.1 mm.

**Figure 14:**
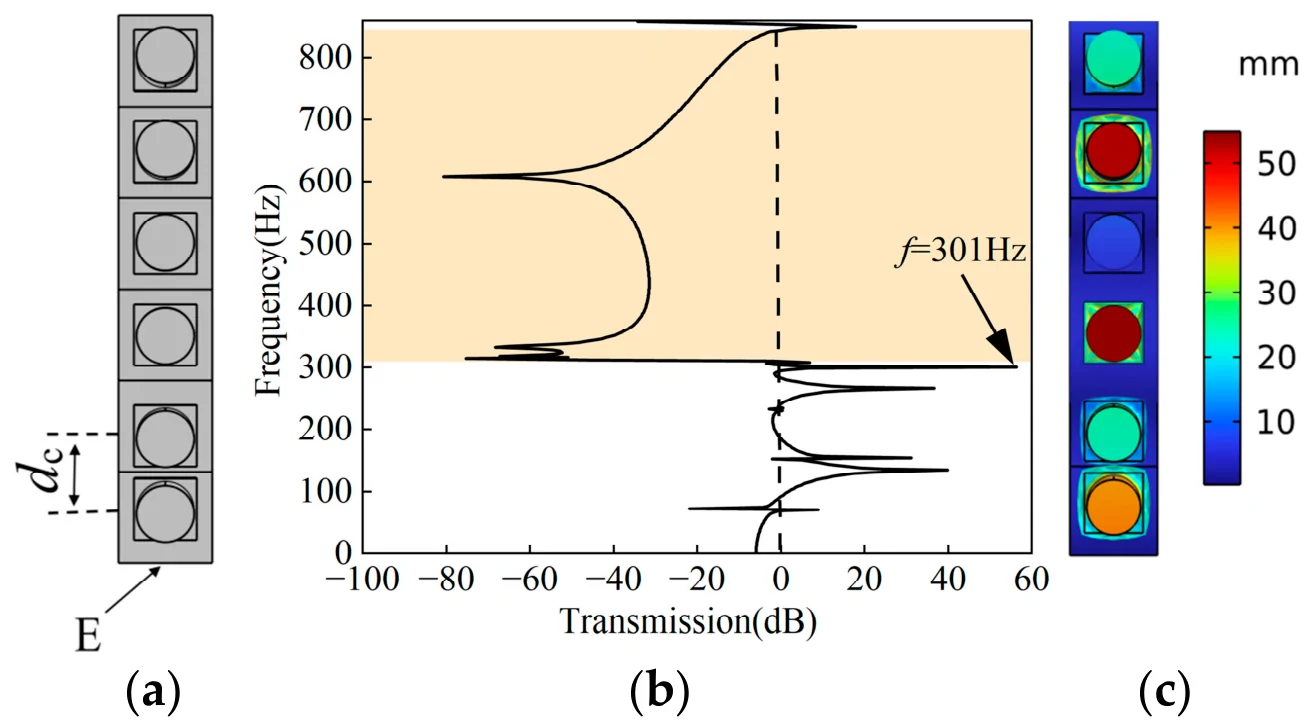
PMMB with a spacing defect of *d_c_* = 12 mm between the 1st and 2nd PLRs: (**a**) top view; (**b**) transmission spectrum; (**c**) top view of vibration mode shape at 301 Hz. This reduced spacing is achieved by moving the two PLRs toward each other. Excitation: *u*_1_ = 0.1 mm.

**Figure 15:**
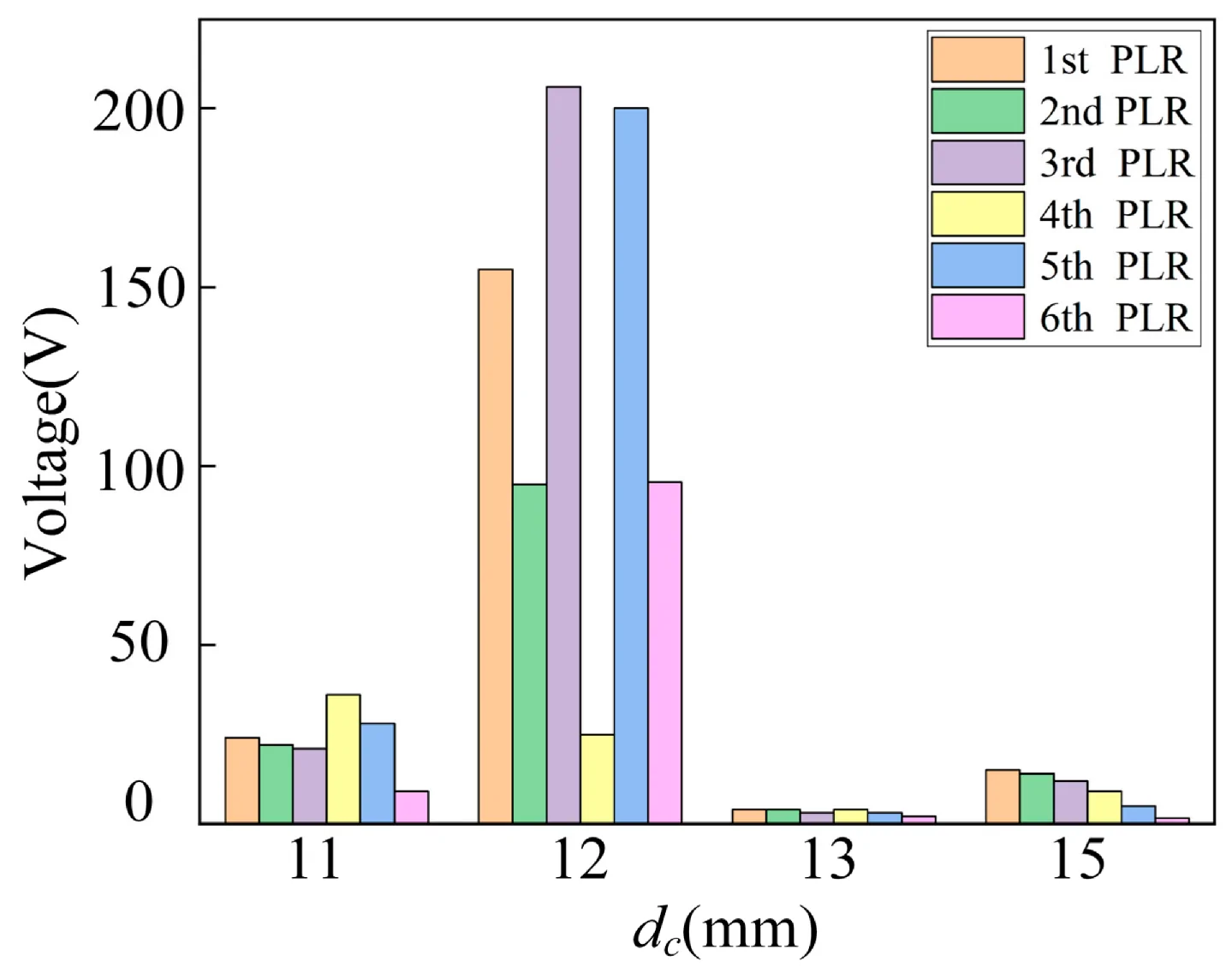
Maximal open-circuit voltage outputs of six PLRs in PMMB with various spacing between the 1st and 2nd PLRs. Excitation: *u*_1_ = 0.1 mm.

**Figure 16:**
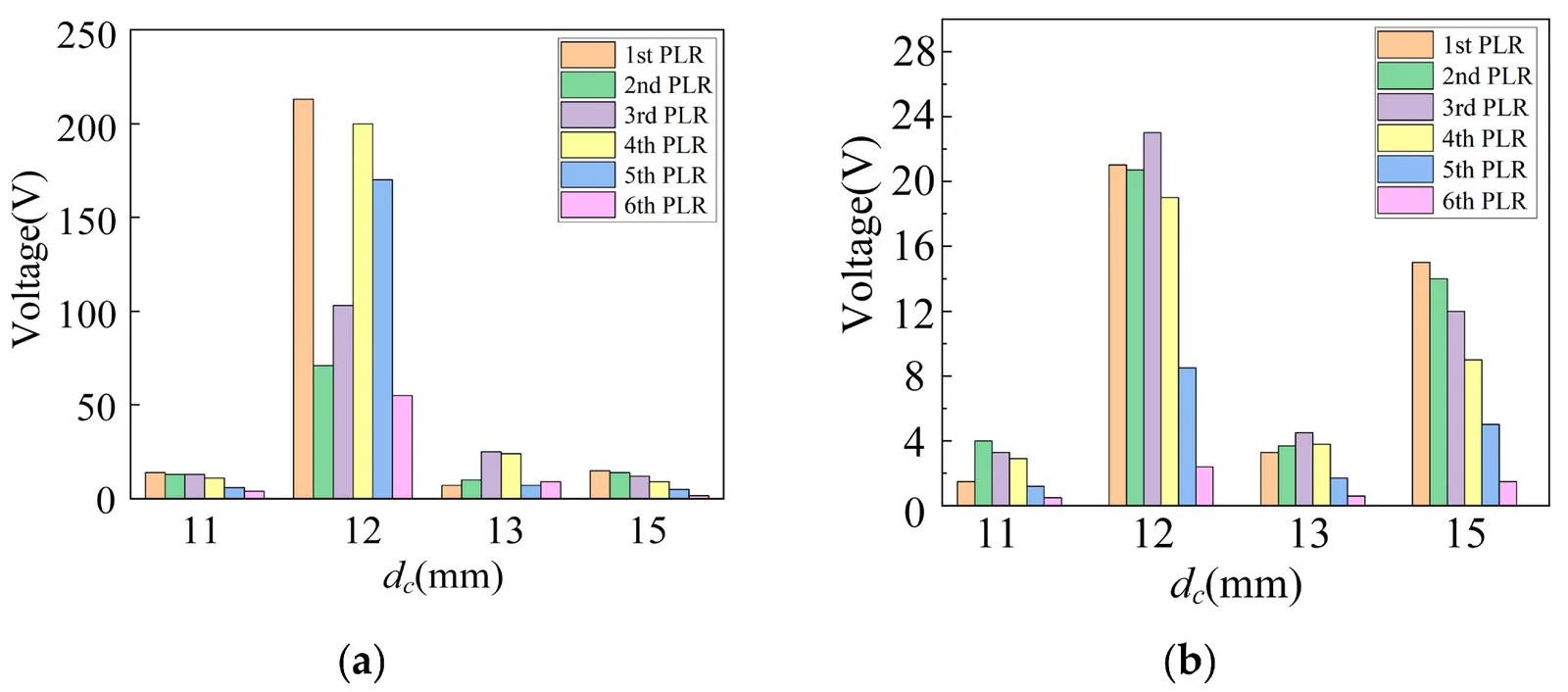
Maximal open-circuit voltage outputs of six PLRs in PMMB for different spacing defects: (**a**) between the 2nd and 3rd PLRs; (**b**) between the 3rd and 4th PLRs. Excitation: *u*_1_ = 0.1 mm.

**Figure 17:**
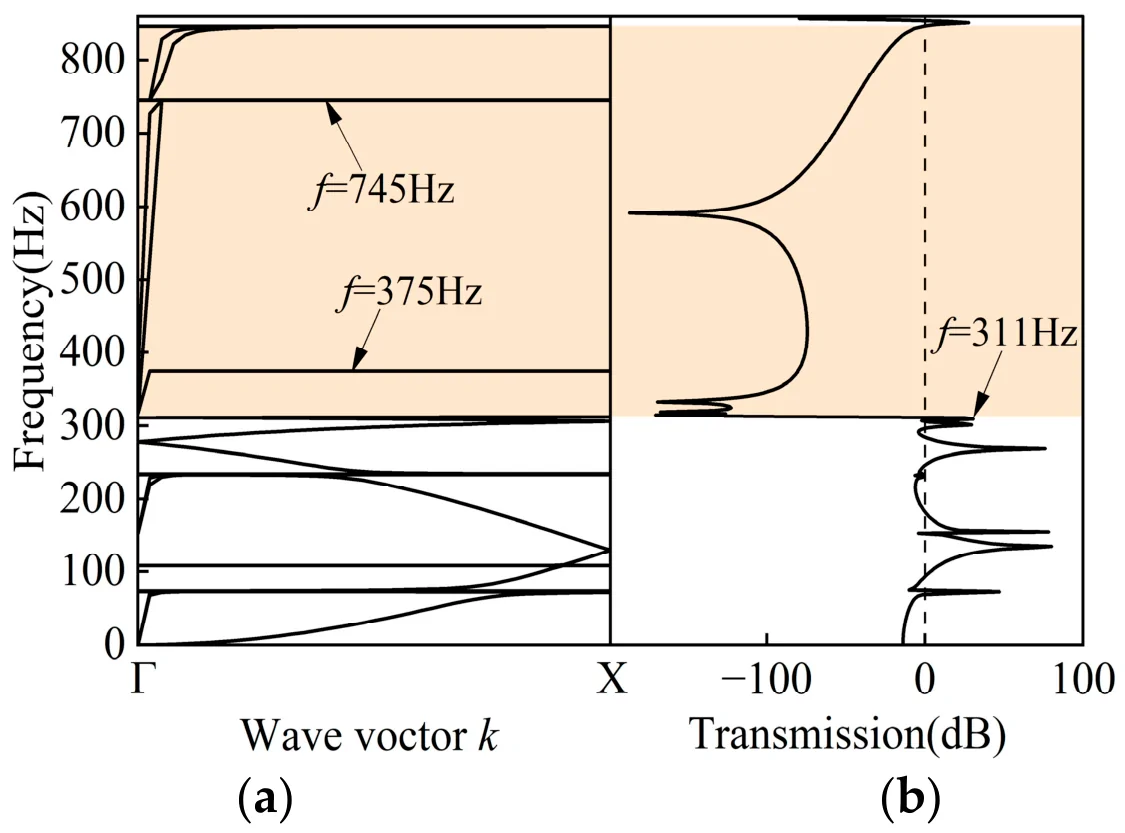
(**a**) Dispersion curves and (**b**) transmission spectrum for the PMMB with an electrical defect created by connecting a 12 H inductor to the 3rd PLR.

**Figure 18:**
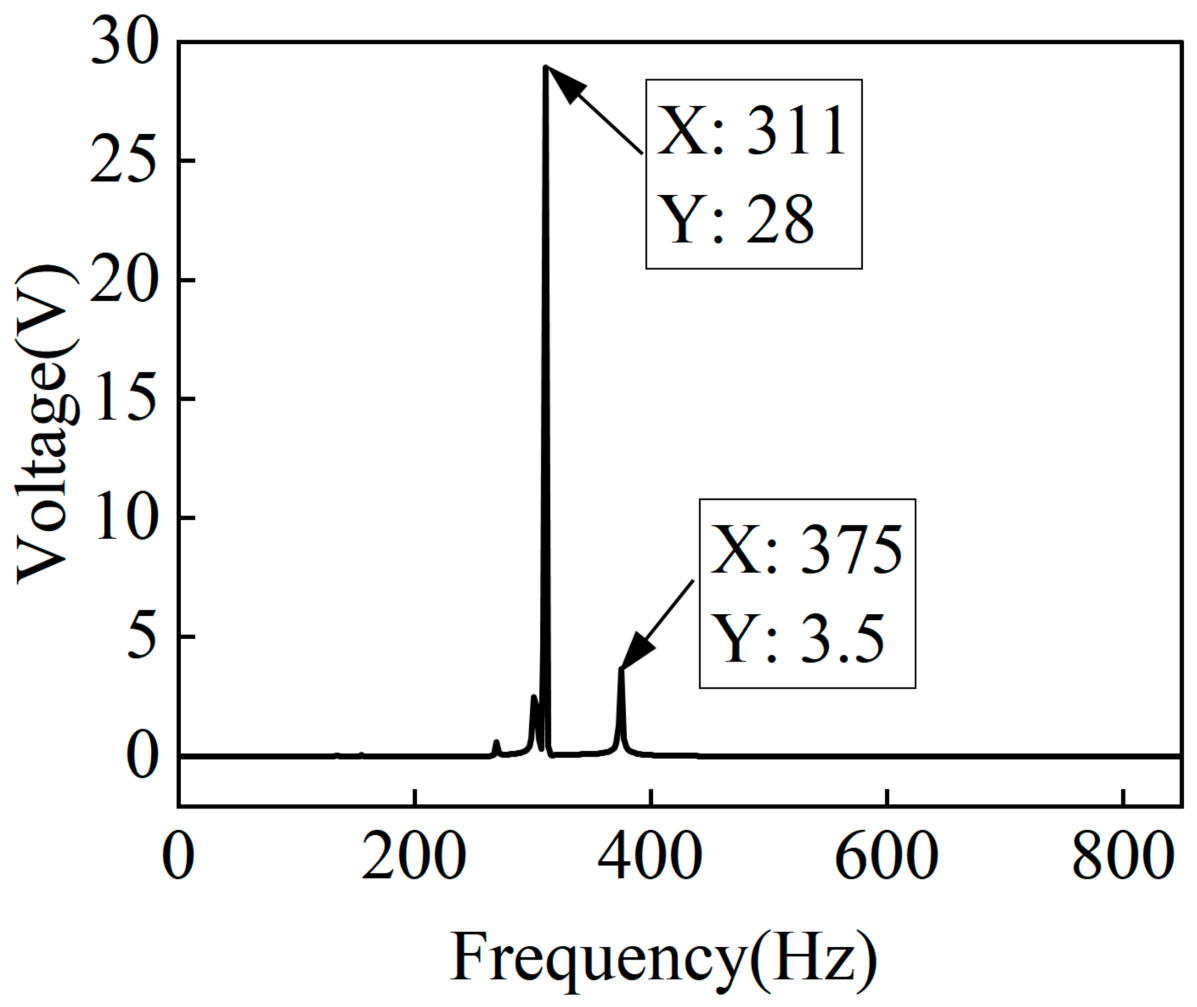
Open-circuit voltage output of the 3rd PLR in the PMMB with an electrical defect created by connecting a 12 H inductor to it. Excitation: *u*_1_ = 0.1 mm.

**Figure 19:**
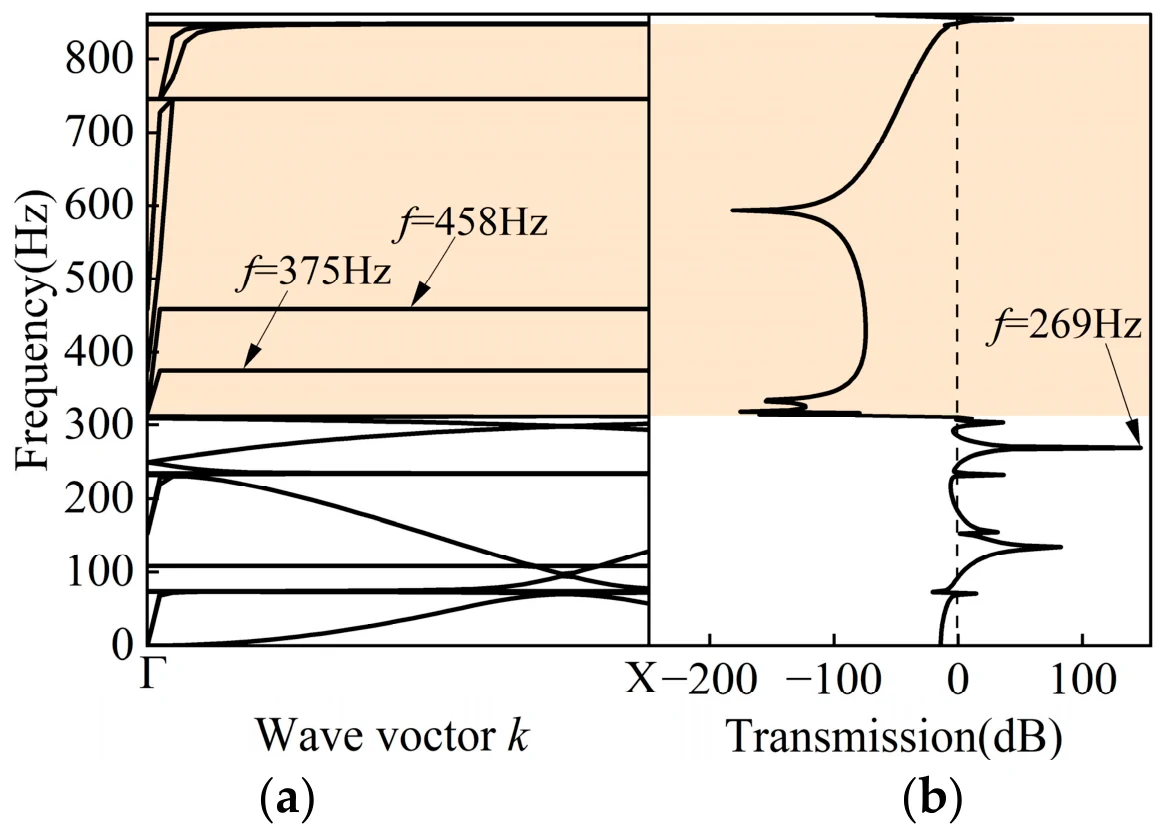
(**a**) Dispersion curves and (**b**) transmission spectrum for the PMMB with electrical defects created by connecting a 12 H inductor to the 3rd PLR and an 8 H inductor to the 5th PLR.

**Figure 20:**
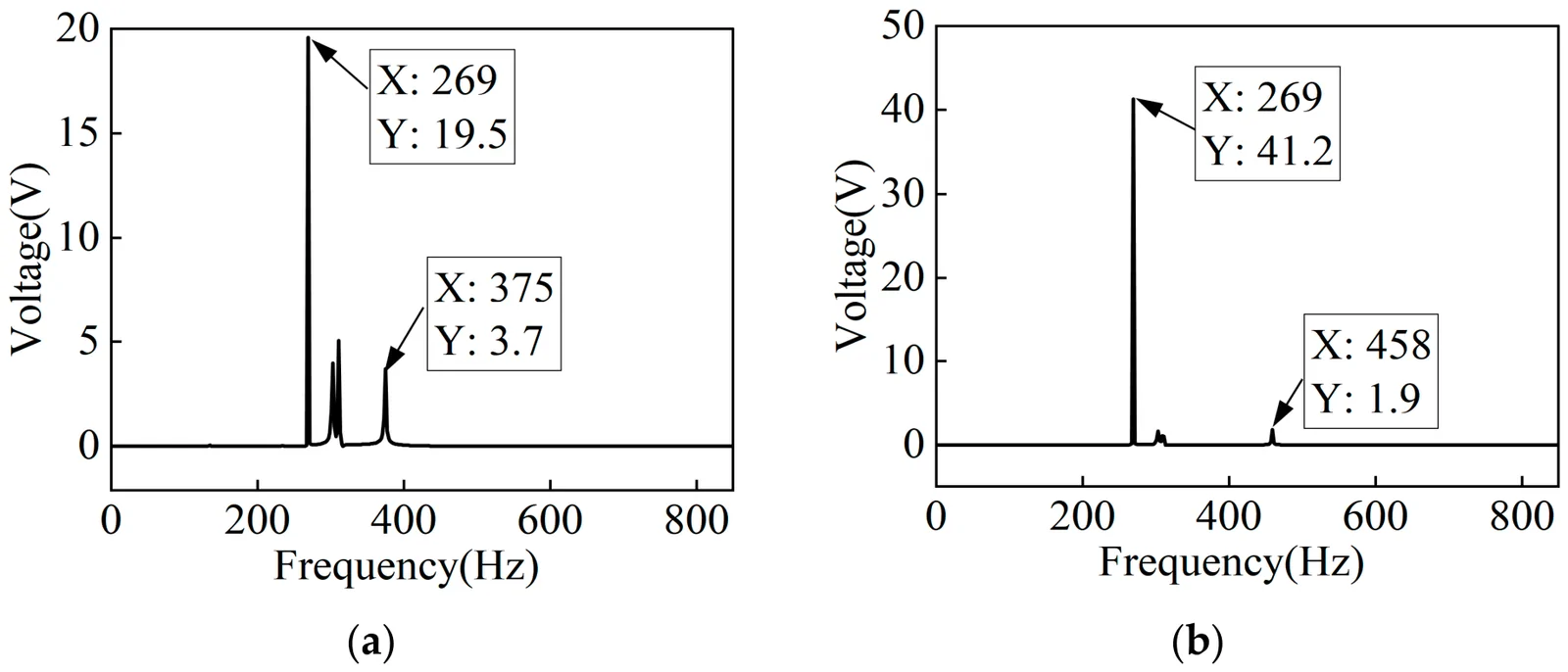
Open-circuit voltage outputs of the 3rd and 5th PLRs in the PMMB with electrical defects: (**a**) with a 12 H inductor on the 3rd PLR; (**b**) with an 8 H inductor on the 5th PLR. Excitation: *u*_1_ = 0.1 mm.

**Table 1:** Material properties of the unit cell of a PMMB.

Material	Mass Density (kg/m^3^)	Elastic Modulus (GPa)	Compliance Constants (10^−12^ m^2^/N)	Poisson’s Ratio	Piezoelectric Strain Constants (10^−12^ C/N)	Relative Permittivity
Aluminum substrate	2700	70	—	0.3	—	—
Rubber plate	1300	1.175 × 10^−4^	—	0.47	—	—
Steel stub	7800	200	—	0.33	—	—
Piezoelectric plate (PZT-5H)	7500	—	*s*_11_ = 16.5 *s*_12_ = −4.78 *s*_13_ = −8.45 *s*_33_ = 20.7 *s*_44_ = 43.5 *s*_66_ = 42.6	—	*d*_31_ = −274 *d*_33_ = 593 *d*_15_ = 741	$\varepsilon_{11}^{T}/\varepsilon_{0}$ = 3130 $\varepsilon_{33}^{T}/\varepsilon_{0}$ = 3400

**Table 2:** Comparison of adjacent and farthest PLR voltages, lower effective BG edge, summed voltage, and energy localization for spacing defects at *d_c_* = 12 mm.

Position Change PLRs	Adjacent PLRs (Avg. Voltage, V)	Farthest PLRs (Avg. Voltage, V)	Ratio (Adj./Far.)	Lower Edge of Effective BG (Hz)	Major Peak Frequency (Hz)	Summed Voltage ^1^ (V)	Degree of Energy Localization
1st–2nd	124.8 (1st, 2nd)	147.9 (5th, 6th)	0.84	311	301	775.8	None
2nd–3rd	87.7 (2nd, 3rd)	116.6 (5th, 6th)	0.75	311	309	732.8	None
3rd–4th	21.1 (3rd, 4th)	11.7 (1st, 6th)	1.81	311	311	92.6	Weak
Perfect PMMB	—	—	—	311	311	58.5	—

**^1^** Summed voltage is the arithmetic sum of the six PLR voltages at the major peak frequency, used as a theoretical indicator of global energy output.

**Table 3:** Comparison of key results for different defect types in the PMMB.

Defect Type	Optimal Parameter	Harvesting PLR	Peak Voltage (V)	Effective BG Variation	Physical Mechanism
Mass defect (stub density change)	*ρ*_3_ = 2700 kg/m^3^	3rd PLR	23.56 (at 437 Hz)	Narrowed	Local mass change → localized defect state inside the effective BG
Spacing defect * (substrate length change)	*d_c_* = 45 mm (3rd–4th PLRs)	PZT patch on beam between 3rd and 4th PLRs	25 (at 655 Hz)	Narrowed	Local stiffness change → localized defect state inside the effective BG
Spacing defect * (PLR position change)	*d_c_* = 12 mm (1st–2nd PLRs)	All six PLRs	775.8 (summed voltage at 301 Hz)	Unchanged	Defect–boundary interference → global energy redistribution across all six PLRs in passband
Electrical defect (single inductor)	*L*_3_ = 12H (3rd PLR)	3rd PLR	28 (at 311 Hz)	Unchanged	LC resonance → voltage response at lower effective BG edge (major peak)
Electrical defect (dual inductors)	*L*_3_ = 12H, *L*_5_ = 8H (3rd, 5th PLRs)	3rd, 5th PLRs	19.5, 41.2 (at 269 Hz)	Unchanged	LC resonance → voltage responses in passband (major peaks)
Perfect PMMB	—	1st PLR	15.28 (at 311 Hz)	BG: 311–850 Hz	Baseline reference

* Spacing defect (substrate length change): length-increased substrate between two adjacent PLRs; spacing defect (PLR position change): adjacent PLRs moved toward each other.

## Data Availability

The original contributions presented in this study are included in the article. Further inquiries can be directed to the corresponding author.
